# The effect of laparoscopic vertical sleeve gastrectomy and laparoscopic roux-en-Y gastric bypass on gastroesophageal reflux disease: An updated meta-analysis and systematic review of 5-year post-operative data from randomized controlled trials

**DOI:** 10.1007/s00464-024-11303-x

**Published:** 2024-10-09

**Authors:** Muhammed Ashraf Memon, Emma Osland, Rossita Mohamad Yunus, Zahirul Hoque, Khorshed Alam, Shahjahan Khan

**Affiliations:** 1 School of Mathematics, Physics and Computing and Centre for Health Research, University of Southern Queensland, Toowoomba, QLD Australia; 2 Sunnybank Obesity Centre & South East Queensland Surgery (SEQS), Sunnybank, QLD Australia; 3 Mayne Medical School, School of Medicine, University of Queensland, Brisbane, QLD Australia; 4 Faculty of Health Sciences and Medicine, Bond University, Gold Coast, QLD Australia; 5 Faculty of Health and Social Science, Bolton University, Bolton, Lancashire UK; 6 Department of Dietetics and Food Services, Royal Brisbane and Women’s Hospital, Herston, QLD Australia; 7 Department of Human Movements and Nutrition, University of Queensland, Brisbane, QLD Australia; 8 Institute of Mathematical Sciences, Universiti Malaya, Kuala Lumpur, Malaysia; 9 School of Mathematics, Physics and Computing, University of Sourthern Queensland, Toowoomba, QLD Australia; 10 School of Business & Centre for Health Research, University of Southern Queensland, Toowoomba, QLD Australia; 11 School of Science and Engineering, Asian University of Bangladesh, Dhaka, Bangladesh

**Keywords:** Bariatric surgery, Gastroesophageal reflux disease, Laparoscopic, Roux-en-Y gastric bypass, Sleeve gastrectomy, Meta-analysis, Systematic review

## Abstract

**Background:**

To evaluate 5-year effect of laparoscopic vertical sleeve gastrectomy (LVSG) versus laparoscopic roux-en-Y gastric bypass (LRYGB) on gastroesophageal reflux disease (GERD) solely based on randomized controlled trials (RCTs).

**Methods:**

A systematic review and meta-analysis of 5-year postoperative GERD data comparing LVSG and LRYGB in adults were undertaken. Electronic databases were searched from January 2015 to March 2024 for publications meeting inclusion criteria. The Hartung–Knapp–Sidik–Jonkman random effects model was applied to estimate pooled odds ratio where meta-analysis was possible. Bias and certainty of evidence were assessed using the Cochrane Risk of Bias Tool 2 and GRADE.

**Results:**

Five RCTs were analysed (LVSG *n* = 554, LRYGB *n* = 539). LVSG was associated with increased adverse GERD outcomes compared to LRYGB at 5 years. The odds for revisional surgery to treat GERD in LVSG patients were 11 times higher compared to LRYGB (OR 11.47, 95% CI 1.83 to 71.69; *p* = 0.02; *I*^2^ = 0% High level of certainty). Similarly pharmacological management for increasing GERD was significantly more frequent in LVSG patients compared to LRYGB (OR 3.89, 95% CI 2.31 to 6.55; *p* ≤ 0.01; *I*^2^ = 0% Moderate level of certainty). Overall, LVSG was associated with significantly more interventions (both medical and surgical) for either worsening GERD and/or development of de novo GERD compared to LRYGB (OR 5.98, 95% CI 3.48 to 10.29; *p* ≤ 0.01; *I*^2^ = 0%) Moderate level of certainty).

**Conclusions:**

The development and worsening of GERD symptoms are frequently associated with LVSG compared to LRYGB at 5 years postoperatively requiring either initiation or increase of pharmacotherapy or failing that revisional bariatric surgery. Appropriate patient/surgical selection is crucial to reduce these postoperative risks of GERD.

**Supplementary Information:**

The online version contains supplementary material available at 10.1007/s00464-024-11303-x.

There exists a strong correlation between obesity (as defined by raised body mass index) and GERD symptoms, oesophageal acid exposure and GERD complications such as reflux esophagitis (RE), erosive esophagitis (EE), Barrett’s esophagitis (BE), and oesophageal adenocarcinoma (EAC). This has been confirmed in a number of population-based studies conducted over the last couple of decades [1–3]. Hampel et al. [4] conducted a systematic review and meta-analysis of 9 epidemiological studies that examined the association between BMI and several GERD-related disorders. Six of 7 studies found significant associations of BMI with EE, 6 of 7 found significant associations with EAC, and 4 of 6 found significant associations with gastric cardia adenocarcinoma. The relationship between GERD and obesity is thought to be multifactorial; however, it is generally attributed to an increase in abdominal and intragastric pressure, the presence of hiatal hernia (HH), increased gradient of abdominal to thoracic pressures; the gastroesophageal pressure gradient (GEPG) [5], lower oesophageal sphincter (LES) abnormalities such a hypotensive lower oesophageal sphincter [6], increase frequency of transient lower oesophageal sphincter relaxation (TLESR) [7], and the presence of oesophageal dysmotility although other considerations such as increase oestrogen levels may also play a role in its causation [8].

Bariatric surgery has been demonstrated to be an efficient approach to improve individual obesity-related health outcomes [9]. According to the International Federation for Surgery for Obesity and Metabolic Disorder (IFSO) 8th Global Registry Report published in 2023 [10], 480,970 bariatric procedures were performed in the years 2021 and 2022. The two most common procedures being laparoscopic vertical sleeve gastrectomy (LVSG) (60.4%) and laparoscopic Roux-en-Y gastric bypass (LRYGB) (29.5%). Although both procedures are considered to be effective in producing long-term weight loss and improving comorbidities, several studies have shown worse GERD outcomes following LVSG, and caution has been advocated in performing LVSG in patients with pre-existing severe GERD or BE [11]. We, therefore, undertook a systematic review and a meta-analysis to evaluate 5-year GERD outcomes following LVSG vs LRYGB based on five RCTs [12–16]. The current work represents an update of our previous analysis (PROSPERO CRD42018112054) [17] in the context of a recently published large RCT [16] on this topic.

## Materials and methods

### Search strategies and data collation

**Fig. 1:**
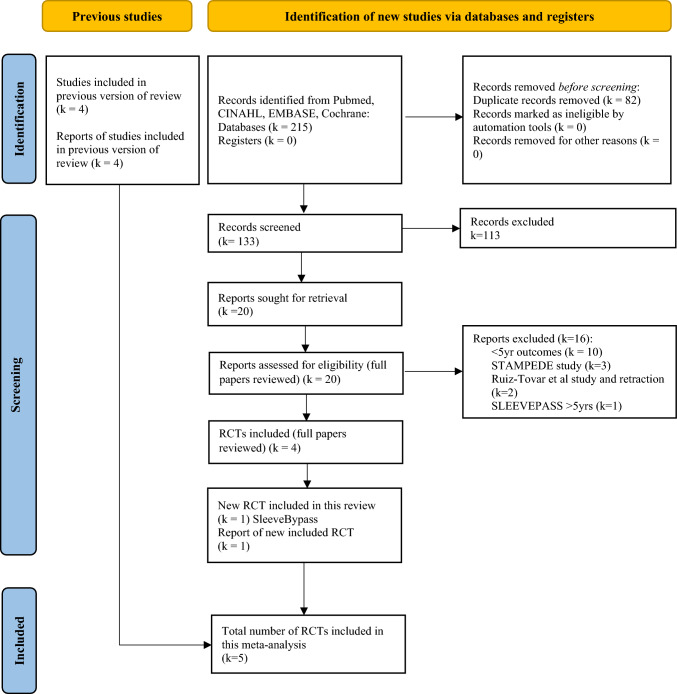
PRISMA flow chart

**Fig. 2:**
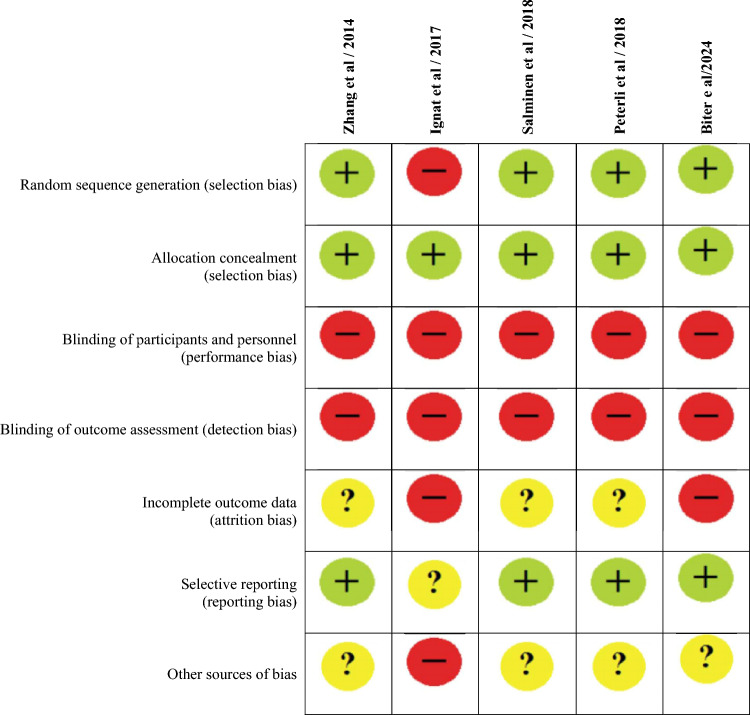
Cochrane’s Risk of bias assessment tool

Electronic databases (Medline, PubMed, EMBASE, Cochrane Register of Systematic Reviews, Science Citation Index) were searched extensively to identify RCTs comparing LVSG and LRYGB (Fig. 1). The search terms were selected for each search engine to optimize and identify all published papers that met the inclusion criteria. Search strategies utilized included combinations of “laparoscopy”[MeSH Terms] OR “laparoscopy”[All Fields] OR “laparoscopic"[All Fields]), “gastric sleeve”[All Fields] OR “sleeve gastrectomy” OR “vertical sleeve gastrectomy” [All Fields], “gastric roux-en-y gastric bypass”[All Fields] OR “gastric bypass,” “gastroesophageal reflux disease”[All Fields] OR “gastro-oesophageal reflux disease”[All Fields], “weight loss surgery”[All Fields] “bariatric surgery”[All Fields], “manometry”[All Fields], “lower esophageal OR oesophageal sphincter”[All Fields], “esophageal OR oesophageal function”[All Fields], “esophageal OR oesophageal motility disorder”[All Fields], “esophageal OR oesophageal motor disorder”[All Fields] “esophageal OR oesophageal dysmotility”[All Fields], “outcomes”[All Fields], “randomised OR randomized controlled trials”[All Fields] AND “comparative trials”[All Fields]. The reference lists of all the retrieved articles were examined for additional citations. Two author (MAM and EO) conducted a literature search and selected records that confirmed compliance with the inclusion criteria. Both authors extracted data from selected studies. The data were compared, and consensus was achieved through discussion or contact with corresponding authors when required. The Cochrane Risk of Bias Tool 2 (RoB2) was applied to included studies (Fig. 2) [18], and the strength of evidence of the outcomes was assessed using GRADE [19] with the assistance of GRADEPro software [20]. The review has been reported in accordance with the Preferred Reporting Items for Systematic Reviews and Meta-Analyses (PRISMA) [21].

### Inclusion criteria

Type of Studies: Randomized Controlled Trials in full peer-review journals.

Publication dates: January 1999 to March 2024.

Type of Intervention: LVSG vs LRYGB.

Type of participants: Morbidly obese adults (≥ 18 years).

Outcome measured: GERD.

Sample Language: English.

### Exclusion criteria

Non-human studies, duplicate studies, abstracts, conference articles, opinion pieces, editorial letters, case studies, reviews, and meta-analyses were excluded from the final review.

### Statistical analysis

All included studies underwent qualitative and quantitative analyses for variables where sufficient data were available. The pooled odds ratio (OR) was used to measure the association between binary outcome using the Hartung–Knapp–Sidik–Jonkman (HKSJ) estimation method for random effects model (REM) [22]. Heterogeneity was assessed using Cochrane’s *Q* statistic and *I*^2^ index [23]. Point estimates of the population effect sizes and forest plots of 95% confidence intervals were produced using meta for package in R [24]. Funnel plots were generated to assess the presence of publication bias [25, 26]. Test of significance of the population effect size was conducted using z-statistic. A *p* value of ≤ 0.05 was considered to be statistically significant.

## Results

**Fig. 3:**
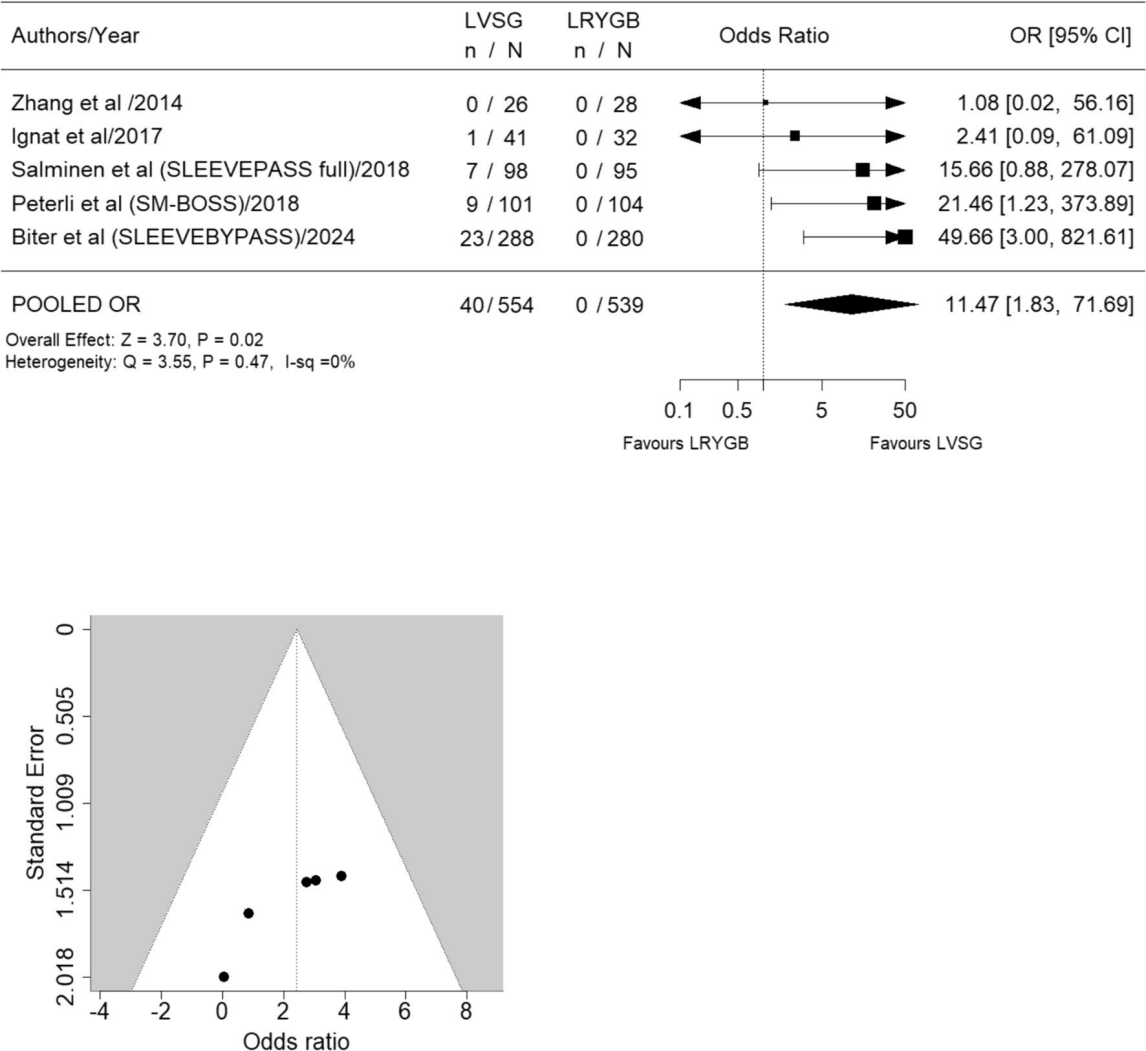
Forest and funnel plots for revisional surgery for GERD

**Fig. 4:**
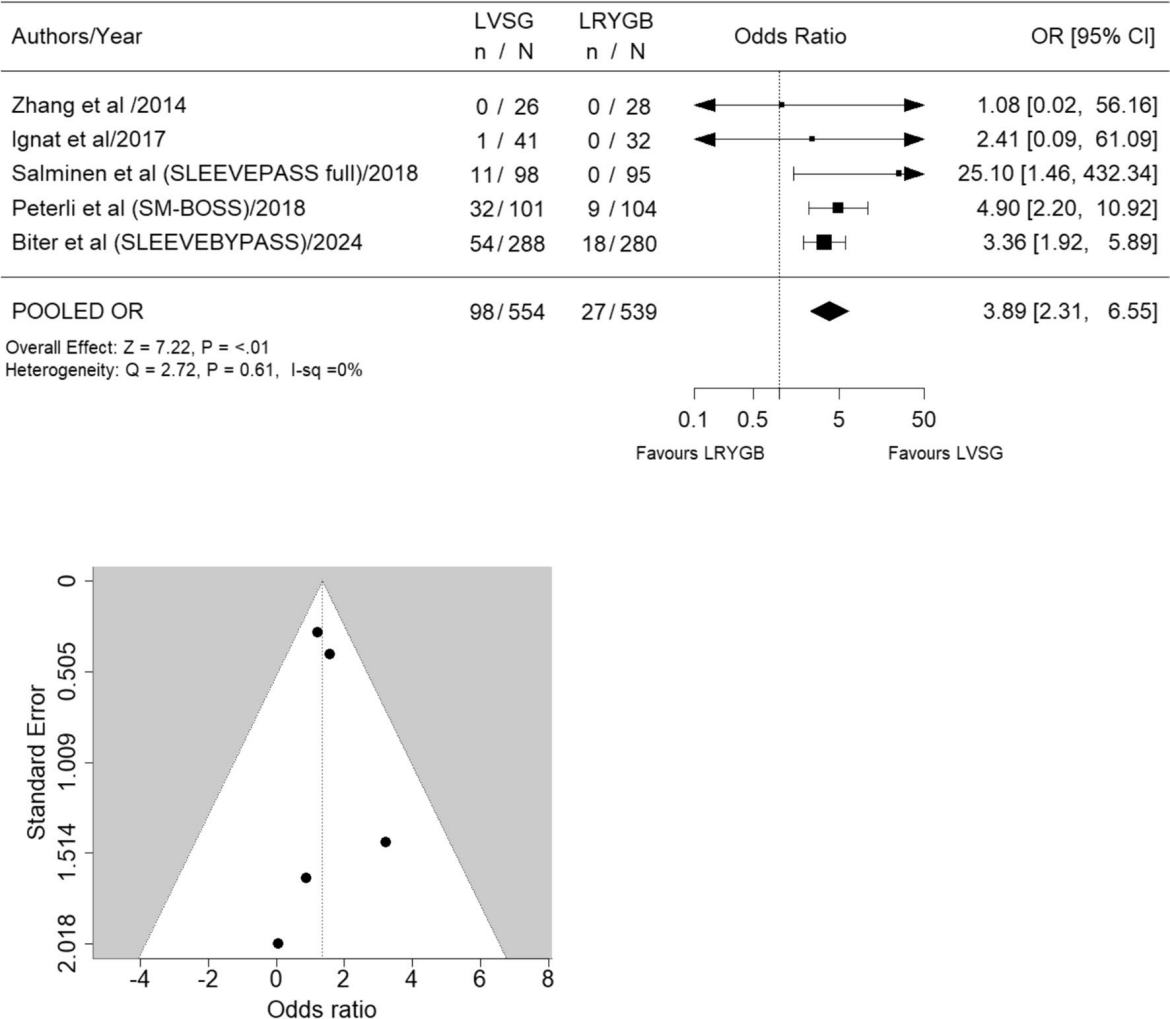
Forest and funnel plots for medical management for GERD

**Fig. 5:**
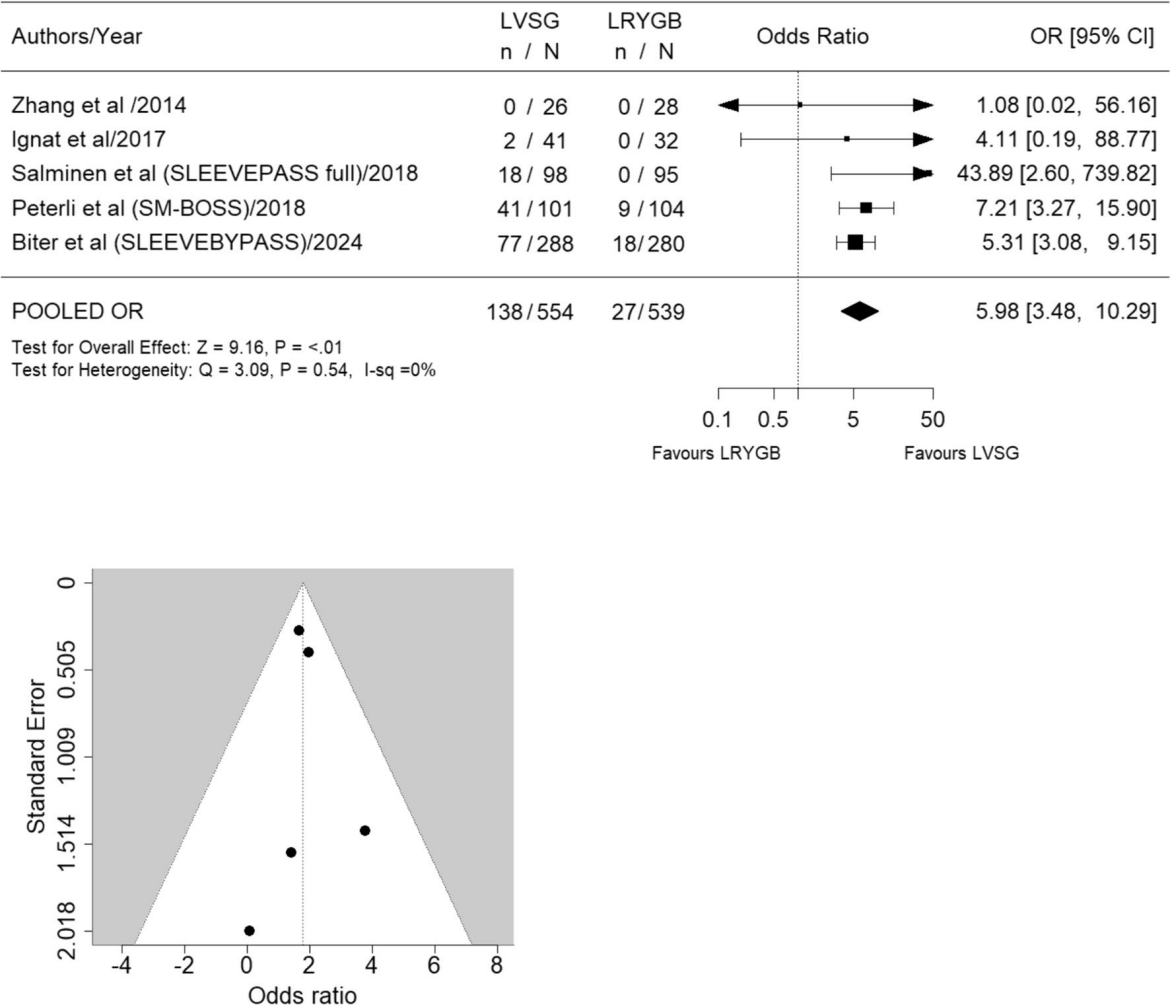
Forest and funnel plots for all treatment modalities for GERD

Eight studies [12–16, 27–29] meeting the inclusion criteria were identified; however, only five studies [12–16] were included in the final analysis (LVSG *n* = 554, LRYGB *n* = 539) (Fig. 1, Table 1). Excluded studies included (a) SLEEVEPASS study [27] reporting 5- to 7-year data which fell outside of the specified 5-year timeframe (b) Ruiz-Tovar et al. study [28], due to errors in data transcription leading to its retraction and (c) The STAMPEDE study [29] due to the addition of intensive medical interventions alongside surgical interventions representing a significant confounding factor. All included studies reported 5-year follow-up data on GERD [12–16]. Revisional surgery was significantly more frequent for GERD treatment in LVSG patients (OR 11.47, 95% CI 1.83 to 71.69; *p* = 0.02; *I*^2^ = 0%) (Fig. 3). Similarly pharmacological management for increasing GERD was significantly more common in LVSG patients compared to LRYGB (OR 3.89, 95% CI 2.31 to 6.55; *p* ≤ 0.01; *I*^2^ = 0%) (Fig. 4). LVSG was associated with significantly more intervention (both medical and surgical) for either worsening GERD and/or development of de novo GERD compared to LRYGB (OR 5.98, 95% CI 3.48 to 10.29; *p* ≤ 0.01; *I*^2^ = 0%) (Fig. 5). A moderate to high level of bias with relation to GERD outcomes was seen in all five studies (Supplementary Material 1) and the certainty of evidence ranged from moderate to high (Table 2). Funnel plots (Figs. 3, 4, 5) did not suggest the presence of publication bias; however, they may have been underpowered to detect this due to small number of studies [26].

**Table 1:** Salient features of included RCTs

Author/year/country/(study acronym)	SC or MC/duration/year started)/trial identifier	Patients at baseline		Patients at 5 years F/U		Contribution of each study at 5 years	Bougie size	DFP	Revision for SS	Type of analysis	Inclusions			Exclusions	GERD outcomes described
		LVSG	LRYGB	LVSG	LRYGB	LVSG + LRYGB	LVSG	LVSG			BMI	Age	Other		
		*n*	*n*	*n* (%)	*n* (%)	*n* (%)	Fr	cm	*n*		kg/m^2^	years			
Zhang et al./2014/China	SC/5 years (2007)/no trial number	32	32	26 (87.5)	28 (81.2)	54 (4.94%)	34	5	0	ITT (superiority)	> 32 to < 50	16 to 60	Acceptance of randomization	Chronic or psychiatric illness, substance abuse, previously GI surgery	*Comorbidity* Not reported as comorbidity *Complication* GERD-related complications reported
Ignat et al./2017/France	SC/non-inferiority trial; 10 years/(2009)/NCT02475590	55	45	32 (71.1)	41 (75.4)	73 (6.67%)	36	5–6	1	ITT (superiority)	> 40 and < 60	18 to 60	No CI for surgery or anaesthesia, able and willing to provide consent	Psychiatric illness, pregnancy, immune- suppression, coagulopathy, anaemia, malabsorptive disease, MI, angina or HF, previous GI surgery, HH > 2 cm	*Comorbidity* Not reported as comorbidity *Complication* Hospital readmission and/or surgical intervention for GERD-related complications
Salminen et al./2018/Finland (SLEEVEPASS)	MC/3 sites/equivalence trial/15 years/(2008)/NCT00793143	121	119	98 (80.1)	95 (81.1)	193 (17.6%)	33–35	4–6	0	PP (equivalence— ± 9% EWL)	≥ 40 or ≥ 35 with comorbidities	18 to 60	Previously failed conservative management	BMI > 60, psychiatric illness, eating disorder, alcohol or substance abuse, active gastric ulcer disease, severe GERD with large HH, previous bariatric surgery	*Comorbidity* Not reported as comorbidity *Complication* Major (Clavien-Dindo IIIb or above) and minor (Clavien-Dindo IIIa or below) GERD-related complications
Peterli et al./2018/Switzerland (SM-BOSS)	MC/4 sites/5 years (2006)/NCT00356213	101	104	101 (90.1)	104 (92.8)	205 (18.7%)	35	3–6	1	PP (equivalence— ± 10% EBMIL)	> 40 with comorbidities	18 to 65	2 + years unsuccessful conservative management	Major abdominal surgery, IBD, previous bariatric surgery, severe GERD despite medication, large HH, expected dense small bowel adhesions	*Comorbidity* Remission, improved, unchanged or worsened from baseline or d*e novo* development as defined by physician at each review *Complication* Reoperation or intervention for GERD-related complications
Biter et al./2024/The Netherlands/(SleeveBypass)	MC/2 sites/5 years (2012) Protocol No. 2011–48	312	316	247 (79.1)	239 (75.6)	568 (51.96%)	36	6	2	ITT (superiority)	> 40	43 ± 11	Acceptance of randomization No CI for surgery or anaesthesia, able and willing to provide consent All patients suitable for metabolic surgery according to international guidelines	Severe GERD cannot be managed without PPIs, know symptomatic HH, prior metabolic or major abdominal surgery and inability to provided informed consent or understanding the questionnaires	*Comorbidity* Resolution, partial resolution, no difference, worsened from baseline or de novo development *Complication* Major ≥ Clavien-Dindo IIIa

BMI = Body Mass Index; CI = Contraindication; DFP = Distance from pylorus; EBMIL = Excess BMI loss; EWL = Excess weight loss; Fr = French; F/U = Follow-up; GERD = Gastroesophageal reflux disease; GI = Gastrointestinal; HbA1C = Glycosylated haemoglobin; HDL-C = High density lipoprotein cholesterol; HF = Heart failure; HH = hiatus hernia; HTN = Hypertension; LDL-C = Low density lipoprotein cholesterol IBD = Inflammatory Bowel Disease; ITT = Intention to treat; MC = Multicentre; MI = Myocardial infarction; OSA = Obstructive Sleep Apnoea; RCT = Randomized Controlled Trial, RS = Revisional Surgery; SC = Single Centre; SS = Sleeve Stenosis; T2DM = Type 2 Diabetes Mellitus; TG = triglycerides

**Table 2:** GRADE evaluation of 5-year postoperative GERD outcomes LVSG vs LRYGB

Certainty assessment							No. of patients		Effect		Certainty
No. of studies	Study design	Risk of bias	Inconsistency	Indirectness	Imprecision	Other considerations	LVSG	LRYGB	Relative (95% CI)	Absolute (95% CI)	
Worsening or development of new of GERD symptoms—All management (follow-up: 5 years; assessed with: PPI use, worsened symptoms, surgical revision)											
5	Randomized trials	Not serious	Serious^a^	Serious^b^	Serious^c^	Strong association all plausible residual confounding would reduce the demonstrated effect	138/554 (24.9%)	27/539 (5.0%)	OR 5.98 (3.48 to 10.29)	190 more per 1,000 (from 105 to 302 more)	⨁⨁⨁◯ Moderate
Worsened or development of new GERD symptoms—new or increased Rx requirements (follow-up: 5 years; assessed with: PPI, symptoms)											
5	Randomized trials	Serious^d^	Serious^a^	Serious^b^	Serious^c^	Very strong association all plausible residual confounding would reduce the demonstrated effect	98/554 (17.7%)	27/539 (5.0%)	OR 3.98 (2.31 to 6.55)	123 more per 1,000 (from 58 to 207 more)	⨁⨁⨁◯ Moderate
Worsened or development of new GERD requiring surgical intervention (follow-up: 5 years; assessed with: LVSG to LRYGB)											
5	Randomized trials	Not serious	Not serious	Not serious	Serious^c^	Very strong association	40/554 (7.2%)	0/539 (0.0%)	OR 11.47 (1.83 to 71.69)	0 fewer per 1,000 (from 0 to 0 fewer)	⨁⨁⨁⨁ High

*CI* confidence interval, *OR* odds ratio

^a^Significant variation in effect sizes in the three largest studies owing to differences in reporting

^b^GERD definition and GERD at baseline not reported in 16% of participants

^c^Low number of events (< 100) does not meet threshold for certainty around precision

^d^Differences in reporting of this outcome may introduce bias and may underestimate real outcome

### How is postoperative GERD described by various RCTs?

The presence of GERD was suspected in the SM-BOSS study [13] based on postoperative consumption of proton pump inhibitors (PPIs) or the presence of postoperative endoscopic esophagitis and/or abnormal esophageal manometry findings. The use of manometry for detecting GERD is questionable as 24-h esophageal pH study is the gold standard [30]. Similarly, in the SleeveBypass Study [16], GERD was supposed if patients were requiring postoperative PPIs. The remaining three RCTs [12, 14, 15] have described GERD as a complication based on subjective patient assessment utilizing various patients’ questionnaires such as Moorehead-Ardelt QOL, Gastrointestinal Quality of Life Index (GIQLI), Bariatric Analysis and Reporting Outcome System (BAROS) QOL score, etc. In the absence of objective postoperative assessments of GERD (such as 24-h pH study), there remains a lack of clarity around the diagnosis of GERD in patients cohort which has the potential to confound and inflate the reported results.

### Preoperative GERD as a comorbidity and its postoperative consequences

The SM-BOSS [13] and the SleeveBypass [16] studies are the only two studies providing a baseline data on preoperative GERD in their patients. In SM-BOSS study, 45% patients were experiencing GERD preoperatively, although the presence of severe GERD was an exclusion criterion in this study [13]. In this study, remission of GERD postoperatively was significantly higher in the LRYGB compared to LVSG patients (60.4% vs 25%, *p* = 0.002 respectively) at 5 years with an absolute difference of 0.36%; 95% CI − 0.57% to 0.15%; *p* = 0.002. On the other hand, both worsening and de novo development of GERD were significantly higher in the LVSG vs LRYGB patients at five years (31.8% vs 6.3%, *p* = 0.006 and 31.6% vs 10.7%, *p* = 0.01, respectively) [13]. Once again, these statistics are based on subjective patients’ data.

In SleeveBypass study [16], 9.6% patients preoperatively reported mild and non-daily GERD symptoms without the need for medications. Following LVSG and LRYGB, 45.8% and 69.4% patients respectively showed improvement in GERD symptoms at 5 years with an absolute difference of 23.6%; 95% CI − 48.5% to 1.3%; p = 0.07. Furthermore, worsening and de novo GERD was significantly more prevalent in LVSG patients compared to LRYGB patients (33.3% and 16% vs 22.2% and 3.6%, respectively). These findings are also based on subjective assessment of patients’ questionnaires.

### GERD as a postoperative complication and revisional bariatric surgery

All the included studies [12–16] provided data on revisional surgery for GERD at 5 years. Of the patients with worsened GERD reported by the SM-BOSS study [13], 9 patients (8.4%) required conversion from LVSG to LRYGB to manage these symptoms or complications. No patients in the LRYGB group required revisional surgery for GERD [13]. The SLEEVEPASS RCT [14] reported GERD as a late complication (minor 9.1%; major 5.8%) in LVSG at 5 years. Seven patients (6%) in the LVSG group required revision to LRYGB for severe reflux [14]. On the contrary, none of the LRYGB patients reported GERD complications at any time point [14]. It is important to note that patients with severe GERD with large hiatal hernias were excluded from this study. In the SleeveBypass study [16], conversion from LVSG to LRYGB occurred in 23 patients (7.4%) for GERD or GERD in conjunction with weight gain. No LRYGB patient underwent revisional surgery for GERD. Ignat et al. [15] reported two LVSG patient with GERD at 5 years. Only one required a conversion to LRYGB for disabling GERD. Conversely, Zhang et al. [12] did not report any patients with GERD in both groups at 5 year. A total of 40 of 554 (7.22%) LVSG patients remaining in follow-up at 5 years required conversion to LRYGB to manage their severe GERD symptoms. None of the 539 LRYGB patient required any revisional surgery for GERD symptoms. Meta-analysis revealed approximately 11 times increased odds for revisional surgery to manage severe GERD in LVSG patients compared to LRYGB cohort at five years (OR 11.47, 95% CI 1.83, 71.69; *p* = 0.02; High level of certainty) (Fig. 3).

### Postoperative de novo GERD/worsened GERD and requirement for pharmacotherapy

The development of new onset GERD following primary bariatric procedures is defined as de novo GERD. Approximately half of these represent preoperative silent (asymptomatic) GERD, while the remainder are newly developed GERD postoperatively [31]. De novo/worsened pre-existing GERD may require initiation of PPIs or increased dosage and failing that, revisional bariatric surgery. Our analysis demonstrated 17.6% (98/554) of LVSG patients compared to 5% (27/539) of LRYGB patients at 5-year postoperatively reported worsened or de novo GERD requiring pharmacological management. Meta-analysis revealed 4 times higher odds for utilizing postoperative pharmacotherapy to manage severe GERD in LVSG patients compared to LRYGB cohort at five years (OR 3.89, 95% CI 2.31 to 6.55; *p* ≤ 0.01; *I*^2^ = 0%) Moderate level of certainty (Fig. 4).

### Postoperative intervention for worsened or new GERD, i.E. De novo GERD (encompassing all interventions)

When consideration all interventions (i.e. pharmacotherapy and/or surgical intervention), analysis demonstrated that 24.9% (138/554) of LVSG patients compared to 5% (27/539) of LRYGB patients were treated for either worsened or de novo GERD. Meta-analysis revealed 6 times increased odds of either a medical and/or surgical intervention to manage severe GERD in LVSG patients compared to LRYGB cohort at five years postoperatively (OR 5.98, 95% CI 3.48 to 10.29; *p* ≤ 0.01; *I*^2^ = 0%) Moderate level of certainty (Fig. 5).

## Discussion

The incidence of worsening GERD and de novo GERD following LVSG can be as high as 31% depending on the length of the follow-up [13]. This is a major concern as GERD can affect the patient’s quality of life leading to negative impact on physical functioning, mental health, and emotional wellbeing and collectively resulting in poorer social interactions [32, 33]. Moreover, protracted GERD can lead to RE, EE, BE, and EAC requiring more stringent surveillance necessitating additional healthcare resource utilization. Therefore, the cost of ongoing regular pharmacotherapy or requirement for revisional bariatric surgery will continue to climb and will have a negative financial impact on both patients and the healthcare system in the future as the prevalence of LVSG increases. The results of this meta-analysis support these findings.

A recent RCT [16] analysing 628 patients at 5 years has reported high incidence of de novo GERD, i.e. 16% in LVSG patients (*n* = 312) compared to 3.6% in LRYGB patients (*n* = 316). Similarly, more patients experienced worsening of GERD symptoms following LVSG compared to LRYGB; 33.3% vs 22.2%, resulting in 11% of patients in the former group requiring revisional surgery compared to none in LRYGB [16]. The SLEEVEPASS RCT [34] at 10 years likewise has shown worsened GERD symptoms post-LVSG (49%) compared to LRYGB (9%). Furthermore, a significantly higher prevalence of RE was noticed in LVSG vs LRYGB patients; 31% (28 of 91) vs 7% (6 of 85). Yet, BE was comparable between two groups (4%). Moreover, GERD-health-related quality of life scores were significantly worse following LVSG relative to LRYGB at 10 years postoperatively.

While there are a number of other published reviews [35–37] on the topic of GERD comparing LVSG and LRYGB patients, their results are weakened by the inclusion of lower methodological quality studies and wide ranging follow-up intervals which introduces preventable heterogeneity impacting the validity and reliability of their conclusions. In this context, Oor et al. [37] have shown that 16 out of 28 studies reported worsened GERD outcomes following LVSG and a statistically non-significant trend towards GERD in LVSG vs LRYGB within the pooled studies.

There are a number of possible factors/variables which may increase the prevalence of GERD following LVSG. They can be divided into modifiable and non-modifiable factors as follows:

### Modifiable factors

#### Sleeve size

The impact of sleeve size on the degree of intragastric pressure (IGP) is inversely proportional to the diameter of the gastric lumen post-LVSG [38, 39]. The higher the IGP, the higher the risk of GERD [39]. Del Genio et al. [40] using 24-h pH-multichannel intraluminal impedance studies have shown significantly increase postprandial retrograde movements of both acid and non-acid effluent post-LVSG probably due to gastric stasis and postprandial regurgitation. A retrospective analysis on 120 LVSG patients using a 42 Fr vs 32 Fr bougie revealed that 82.1% of patient in the former group compared to 61.1% in the latter group were completely cured of GERD symptoms [41]. In contrast, Weiner et al. [42] failed to show any difference in GERD symptoms two years following LVSG using either a 44 Fr or 32 Fr bougies. Whether just the bougie size in isolation has a major impact on GERD is contentious. All the included studies in our meta-analysis have used small size bougies (Ignat et al. [15] and Biter et al. [16]: 36 Fr bougies; Zhang et al. [12] 34 Fr bougies; SLEEVEPASS [14] 33–35 Fr bougies; SM-BOSS [13] 35 Fr bougies) (Table 1) which in combination with other factors (see below) may be one of the reasons for a high incidence of GERD across these studies.

#### Sleeve shape

Four LVSG shapes have been identified based on radiology studies and include tubular, superior pouch, inferior pouch, and dumbbell shape. Toro et al. [43], when analysing GERD symptoms based on GERD-health-related quality of life score, found larger superior pouches are associated with a significantly higher risk of GERD which mostly likely is due to a large acid secreting area. Keidar et al. [44] similarly have shown that dilated superior pouch is associated with higher incidence of reflux. On the other hand, inferior pouch was associated with least GERD symptoms due to higher antral capacity to distend and accommodate gastric contents [45, 46]. No information is available regarding the shape of gastric sleeves in any of these RCTs due to lack of radiological data.

#### Antral preserving (AP) vs antral resecting (AR)—distance from the pylorus (DFP)

Garay et al. [47] analysed gastric scintigraphy of two groups based on the distance of application of first stapler from the pylorus (DFP). In the AP group, the first stapler firing took place 5 cm from the pylorus, compared with 2 cm from the pylorus in the AR group. A significant accelerated gastric emptying was observed at 2 and 12 months in the antrum preserving group which may therefore decrease the IGP and subsequently GERD symptoms. Pizza et al. [48] similarly have shown increased GER symptoms amongst the AR compared AP group at 12 months follow-up based on GERD-HRQL score. This finding was associated with increased esophagitis on gastroscopy as well as symptoms of food intolerance. A recent meta-analysis [49] addressing the issue of AR vs AP revealed that postoperative GERD at 6 months was significantly lower in the 6 cm DFP group vs 2 cm DFP group. However, this difference disappeared at 12 months postoperatively. The studies represented in this meta-analysis utilized 3 to 6 cm distance for the application of first stapler from the pylorus (SM-BOSS [13]: 3–6 cm; SLEEVEPASS [14]: 4–6 cm; Zhang et al. [12]: 5 cm; Ignat et al. [15]: 5–6 cm; SleeveBypass [16]: 6 cm) (Table 1). There was no subgroup analysis of GERD symptoms for different groups based on the resection distance from the pylorus was available in any of these RCTs.

#### Surgical techniques

Poor surgical techniques resulting in sleeve stenosis (SS), kinking, angulation, twisting and/or cicatrization of the sleeve can lead to increase intragastric pressure (IIGP) and higher risk of GERD. According to D’Alessandro A et al. [50], three different mechanisms may lead to SS which includes (a) inflammatory stenosis due to tissue inflammation which one hope will settle down in time; (b) narrow sleeve due to small bougie (pure stricture) and (c) torsion of the sleeve due to misaligned stapled line (functional stenosis or twist).Therefore, paying attention to surgical technique when creating a sleeve has the potential to decrease the risk of GERD. SS which occurs between 0.5 and 4% [51, 52] around the incisura is associated not only with a higher risk of GERD but other upper gastrointestinal symptoms such as nausea, vomiting, etc. and can also lead to sleeve fistula. The revision rate for SS is around 30% [53]. In the SleeveBypass study [16], 2 patients (5.1%) required revision surgery for symptomatic stenosis, in the SM-BOSS 1 patient [13], in Ignat et al. [15] study 1 gastric twist, and no patients with SS were reported in the SLEEVEASS [14] and Zhang et al. study [12] (Table 1).

### Non-modifiable factors

#### Angle of his (esophagogastric angle)

Quero et al. [54] performed magnetic resonance imaging both pre- and post-LVSG. They reported a more obtuse esophagogastric angle or angle of His in the majority (78%) of patients following LVSG from 36° to 51° which lead to reduction in abdominal and total LES length. Furthermore, EGJ relaxation increased after LVSG, which is associated with decreased intra-abdominal length and resting pressure of the LES. All these anatomical changes lead to reduced viscous resistance to flow across the EGJ leading to a significant increase in GER confirmed with 24-h pH study by a number of authors [55, 56]. Even with the best of surgical techniques to create a perfect sleeve, the widening of the angle of His is usually unavoidable.

#### Lower esophageal sphincter pressure

Several studies have objectively analysed lower esophageal sphincter pressure (LESP) using manometry data [54, 56, 57] and have shown dynamic failure of LES following LVSG leading to increased risk of GERD. This has been attributed to iatrogenic injury of the sling muscle fibre at the cardia while dissecting around the angle of His during the LVSG procedure [58, 59]. It is simply impossible to avoid an iatrogenic injury to the sling fibres during resection of fundus towards the EGJ in the creation of a gastric sleeve.

#### Impact of fundal resection

The effect of resection of the fundus, which is an essential part of LVSG, leads to decreased vasovagal reflex and complete elimination of physiological postprandial gastric relaxation, further increasing IGP which is associated with a higher risk of GERD. Yehoshua et al. [38] undertook volume and pressure assessments pre- and post-LVSG using an electronic barostat. According to their study, the distensibility of the total stomach and excised fundus was ten-fold higher than that of the gastric sleeve, providing a conclusive evidence that the distensible region of the stomach is removed during LVSG, leading to IIGP. Once again, removal of 80–90% of stomach during LVSG is an essential step for this type of restrictive bariatric procedure even if one is utilizing 40–42 Fr bougies.

#### Hiatal hernia (HH)

Several authors have shown that the separation of LES and crural diaphragm (both of which constitute EGJ) occurs more frequently in obese individuals [5, 60, 61]. The consequences of this separation are the disruption of EGJ leading to the formation of the hiatus hernia, the incidence of which varies from 5.4 to 52.6% [60, 61], which leads to disruption of the integrity of the anti-reflux mechanism leading to decrease in the efficacy of the esophageal clearance. Furthermore, the presence of HH also leads to reduction in LESP compared to those without HH (13 vs 8 mmHg) [62] increasing the risk of GERD. HH when combined with high IGP due to stomach reduction, augments the condition for GERD. Repair of ≥ 4 cm HH leads to resolution in 73% of patients with pre-existing GERD symptoms. Furthermore, it prevents the development of de novo GERD [63]. However, information on the size of HH and whether they were repaired routinely has been missing from the included RCTs [12–16].

#### Esophageal dysmotility

Several studies [64–67] have investigated the occurrence and relevance of preoperative esophageal dysmotility in morbidly obese patients using both conventional and high resolution manometry prior to undergoing bariatric surgery. This is because of the growing body of evidence suggesting that morbidly obese patients have an increased prevalence of esophageal motility disorder which can be as high as 61% [64]. The dysmotility can affect both the esophageal body and LOS. Decreased LES pressure combined with ineffective oesophageal motility and IIGP post-LVSG are some of the most likely mechanism exacerbating GER symptoms postoperatively.

#### Limitations

There are several limitations that need to be acknowledged with the present work. First, the low number of RCTs with small patient sample size in 4 out of 5 trials limits statistical power which can lead to both alpha and beta errors and, therefore, impact the reliability and validity of the analysis. Furthermore, a small number of studies also impact the assessment of publication bias with confidence. Nevertheless, this analysis has reported large effect sizes despite the small number of inclusions, and the findings in the 5-year data are supported by the 5 to 10 data that have been recently published, suggesting that these results are robust enough to guide clinical practice. Second, the subjective reporting of GERD limited the impact of the review process, analysis, and the certainty of the evidence. There are, however, a number of irrefutable facts emerged from this meta-analysis which include (a) higher incidence of GERD 5-year post-LVSG compared to LRYGB, (b) higher revision bariatric surgery rates for GERD post-LVSG, and (c) higher pharmacotherapy intervention for worsening or de novo GERD. Similarly, the variation in criteria for diagnosing GERD is a major flaw within and across studies. Likewise, reliance on subjective data/statistics for diagnosing GERD 5-year post-LVSG is also one of the substantial limitations of these RCTs. There a number of other factors which most likely have contributed to GERD in LVSG not reported in these RCTs, including but not limited to early learning curve, shape of the sleeve, effect on angle of His, and preoperative presence of oesophageal dysmotility, all of which we feel have a negative impact on EGJ which is a high pressure protective barrier for prevention of GERD.

## Conclusion

This systematic review and meta-analysis of RCTs have shown that LVSG compared to LRYGB at 5 years postoperatively is associated with significantly increased risk of GERD and de novo GERD requiring increasing pharmacotherapy or revisional bariatric surgery in long term. It is, therefore, imperative that patient selection for bariatric procedure takes into consideration preoperative GERD risk factors. It would seem prudent to incorporate into practice routine preoperative gastroscopy and esophageal function testing prior to offering either LVSG or LRYGB. Likewise, by modifying a number of surgical factors through standardization of operative techniques for LVSG, it is plausible to reduce or eliminate the risk of GERD. This will require a development and adoption of a protocol by the IFSO to standardize the LVSG procedure. This meta-analysis encourages the need for standardization of GERD diagnostic practices and surgical techniques in bariatric patients to minimize the development of GERD and its long-term consequences in the future in LVSG patients.

## Supplementary information

Below is the link to the electronic supplementary material.

Supplementary file1 (DOCX 114 KB)
