# Supplementary material for: The effect of laparoscopic vertical sleeve gastrectomy and laparoscopic roux-en-Y gastric bypass on gastroesophageal reflux disease: An updated meta-analysis and systematic review of 5-year post-operative data from randomized controlled trials
Source: Surg Endosc. 2024 Oct 9;38(11):6254–69. doi: 10.1007/s00464-024-11303-x (PMC11525329; doi:10.1007/s00464-024-11303-x)
Supplement: Supplementary file 1 — Supplementary file1 (DOCX 114 KB) [file 464_2024_11303_MOESM1_ESM.docx]

**Supplementary Material 1: Risk of Bias 2 working by Outcome and Study**

| **Unique ID** | 1 | **Study ID** | 1 | **Assessor** | MAM/EO |
| --- | --- | --- | --- | --- | --- |
| **Author/Ref** | Zhang et al^12^ | **Aim** | assignment to intervention (the 'intention-to-treat' effect) |  |  |
| **Experimental** | LVSG | **Comparator** | LRYGB | **Source** | Journal article(s) |
| **Outcome** | Worsened or de novo GERD | **Results** |  | **Weight** | 1 |
| **Domain** | **Signalling question** | | | **Response** | **Comments** |
| **Bias arising from the randomization process** | 1.1 Was the allocation sequence random? | | | Y | "Computer generated random numbers were used to allocate the type of procedure which was written on a card and sealed completely in an opaque envelope" (p1618) |
|  | 1.2 Was the allocation sequence concealed until participants were enrolled and assigned to interventions? | | | PY |  |
|  | 1.3 Did baseline differences between intervention groups suggest a problem with the randomization process? | | | N | No difference at baseline for reported details - age, gender, BMI (table 1). NB GERD outcomes not described. |
|  | **Risk of bias judgement** | | | **Low** |  |
| **Bias due to deviations from intended interventions** | 2.1.Were participants aware of their assigned intervention during the trial? | | | Y | no blinding of patients, carers, surgeons other clinical staff. |
|  | 2.2.Were carers and people delivering the interventions aware of participants' assigned intervention during the trial? | | | Y |  |
|  | 2.3. If Y/PY/NI to 2.1 or 2.2: Were there deviations from the intended intervention that arose because of the experimental context? | | | PN | Nil reported |
|  | 2.4 If Y/PY to 2.3: Were these deviations likely to have affected the outcome? | | | NA |  |
|  | 2.5. If Y/PY/NI to 2.4: Were these deviations from intended intervention balanced between groups? | | | NA |  |
|  | 2.6 Was an appropriate analysis used to estimate the effect of assignment to intervention? | | | NI |  |
|  | 2.7 If N/PN/NI to 2.6: Was there potential for a substantial impact (on the result) of the failure to analyse participants in the group to which they were randomized? | | | NI |  |
|  | **Risk of bias judgement** | | | **High** | Blinding procedure is difficult to obtain |
| **Bias due to missing outcome data** | 3.1 Were data for this outcome available for all, or nearly all, participants randomized? | | | PN | 18.8% loss to follow up at 5yrs (81 vs 87.5% between groups) |
|  | 3.2 If N/PN/NI to 3.1: Is there evidence that result was not biased by missing outcome data? | | | N |  |
|  | 3.3 If N/PN to 3.2: Could missingness in the outcome depend on its true value? | | | PY | It is reasonable to presume that patients lost to follow up in a bariatric study are lost due at least in part of poor postoperative outcomes, and that those not being followed up at five years will be different to those remaining in follow up. |
|  | 3.4 If Y/PY/NI to 3.3: Is it likely that missingness in the outcome depended on its true value? | | | PY |  |
|  | **Risk of bias judgement** | | | **High** |  |
| **Bias in measurement of the outcome** | 4.1 Was the method of measuring the outcome inappropriate? | | | PN | GERD is not defined and was not specified as an a priori outcome. Should pick up surgical outcomes but not necessarily Rx related. |
|  | 4.2 Could measurement or ascertainment of the outcome have differed between intervention groups? | | | PY | Without a clear definition, detection may vary between clinicians. |
|  | 4.3 Were outcome assessors aware of the intervention received by study participants? | | | NA |  |
|  | 4.4 If Y/PY/NI to 4.3: Could assessment of the outcome have been influenced by knowledge of intervention received? | | | NA |  |
|  | 4.5 If Y/PY/NI to 4.4: Is it likely that assessment of the outcome was influenced by knowledge of intervention received? | | | NA |  |
|  | **Risk of bias judgement** | | | **High** |  |
| **Bias in selection of the reported result** | 5.1 Were the data that produced this result analysed in accordance with a pre-specified analysis plan that was finalized before unblinded outcome data were available for analysis? | | | N | Statistical analysis not stated as being determined prior to study, and no relationship to time prior to unblinding of analysts mentioned. |
|  | 5.2 ... multiple eligible outcome measurements (e.g. scales, definitions, time points) within the outcome domain? | | | PN | Without a definition of GERD, the measure is likely to be subjective. |
|  | 5.3 ... multiple eligible analyses of the data? | | | N | No |
|  | **Risk of bias judgement** | | | **Some concerns** |  |
| **Overall bias** | **Risk of bias judgement** | | | **High** | Main issue stems from GERD not being a priori reported outcome; no defintion of GERD provided. |
|  |  |  |  |  |  |
|  |  |  |  |  |  |
| **Unique ID** | 2 | **Study ID** | 1 | **Assessor** | MAM/EO |
| **Author/Ref** | Zhang et al^12^ | **Aim** | assignment to intervention (the 'intention-to-treat' effect) |  |  |
| **Experimental** | LVSG | **Comparator** | LRGB | **Source** | Journal article(s) |
| **Outcome** | Increased or new need for Rx | **Results** |  | **Weight** | 1 |
| **Domain** | **Signalling question** | | | **Response** | **Comments** |
| **Bias arising from the randomization process** | 1.1 Was the allocation sequence random? | | | Y |  |
|  | 1.2 Was the allocation sequence concealed until participants were enrolled and assigned to interventions? | | | PY |  |
|  | 1.3 Did baseline differences between intervention groups suggest a problem with the randomization process? | | | N |  |
|  | **Risk of bias judgement** | | | **Low** |  |
| **Bias due to deviations from intended interventions** | 2.1.Were participants aware of their assigned intervention during the trial? | | | Y | No blinding of patients, carers, surgeons or other clinical staff. |
|  | 2.2.Were carers and people delivering the interventions aware of participants' assigned intervention during the trial? | | | Y |  |
|  | 2.3. If Y/PY/NI to 2.1 or 2.2: Were there deviations from the intended intervention that arose because of the experimental context? | | | PN | Nil reported |
|  | 2.4 If Y/PY to 2.3: Were these deviations likely to have affected the outcome? | | | NA |  |
|  | 2.5. If Y/PY/NI to 2.4: Were these deviations from intended intervention balanced between groups? | | | NA |  |
|  | 2.6 Was an appropriate analysis used to estimate the effect of assignment to intervention? | | | NI |  |
|  | 2.7 If N/PN/NI to 2.6: Was there potential for a substantial impact (on the result) of the failure to analyse participants in the group to which they were randomized? | | | NI |  |
|  | **Risk of bias judgement** | | | **High** | Blinding procedure is difficult to obtain |
| **Bias due to missing outcome data** | 3.1 Were data for this outcome available for all, or nearly all, participants randomized? | | | PN | 18.8% loss to follow up at 5yrs (81 vs 87.5% between groups) |
|  | 3.2 If N/PN/NI to 3.1: Is there evidence that result was not biased by missing outcome data? | | | N |  |
|  | 3.3 If N/PN to 3.2: Could missingness in the outcome depend on its true value? | | | PY | It is reasonable to presume that patients lost to follow up in a bariatric study are lost due at least in part of poor postoperative outcomes, and that those not being followed up at five years will be different to those remaining in follow up. |
|  | 3.4 If Y/PY/NI to 3.3: Is it likely that missingness in the outcome depended on its true value? | | | PY |  |
|  | **Risk of bias judgement** | | | **High** |  |
| **Bias in measurement of the outcome** | 4.1 Was the method of measuring the outcome inappropriate? | | | PN | GERD is not defined and was not specified as an a priori outcome. Should pick up surgical outcomes but not necessarily Rx related. |
|  | 4.2 Could measurement or ascertainment of the outcome have differed between intervention groups? | | | PY | Without a clear definition, detection may vary between clinicians. |
|  | 4.3 Were outcome assessors aware of the intervention received by study participants? | | | NA |  |
|  | 4.4 If Y/PY/NI to 4.3: Could assessment of the outcome have been influenced by knowledge of intervention received? | | | NA |  |
|  | 4.5 If Y/PY/NI to 4.4: Is it likely that assessment of the outcome was influenced by knowledge of intervention received? | | | NA |  |
|  | **Risk of bias judgement** | | | **High** |  |
| **Bias in selection of the reported result** | 5.1 Were the data that produced this result analysed in accordance with a pre-specified analysis plan that was finalized before unblinded outcome data were available for analysis? | | | N | Statistical analysis not stated as being determined prior to study, and no relationship to time prior to unblinding of analysts mentioned. |
|  | 5.2 ... multiple eligible outcome measurements (e.g. scales, definitions, time points) within the outcome domain? | | | PN | Without a definition of GERD, the measure is likely to be subjective. |
|  | 5.3 ... multiple eligible analyses of the data? | | | N | No |
|  | **Risk of bias judgement** | | | **Some concerns** |  |
| **Overall bias** | **Risk of bias judgement** | | | **High** | Main issue stems from GERD not being a priori reported outcome; no definition of GERD provided. |
|  |  |  |  |  |  |
|  |  |  |  |  |  |
| **Unique ID** | 3 | **Study ID** | 1 | **Assessor** | MAM/EO |
| **Author/Ref** | Zhang et al^12^ | **Aim** | assignment to intervention (the 'intention-to-treat' effect) |  |  |
| **Experimental** | LVSG | **Comparator** | LRGB | **Source** | Journal article(s) |
| **Outcome** | Surgical procedure required | **Results** |  | **Weight** | 1 |
| **Domain** | **Signalling question** | | | **Response** | **Comments** |
| **Bias arising from the randomization process** | 1.1 Was the allocation sequence random? | | | Y |  |
|  | 1.2 Was the allocation sequence concealed until participants were enrolled and assigned to interventions? | | | PY |  |
|  | 1.3 Did baseline differences between intervention groups suggest a problem with the randomization process? | | | N |  |
|  | **Risk of bias judgement** | | | **Low** |  |
| **Bias due to deviations from intended interventions** | 2.1.Were participants aware of their assigned intervention during the trial? | | | Y | no blinding of patients, carers, surgeons or other clinical staff. |
|  | 2.2.Were carers and people delivering the interventions aware of participants' assigned intervention during the trial? | | | Y |  |
|  | 2.3. If Y/PY/NI to 2.1 or 2.2: Were there deviations from the intended intervention that arose because of the experimental context? | | | PN | Nil reported |
|  | 2.4 If Y/PY to 2.3: Were these deviations likely to have affected the outcome? | | | NA |  |
|  | 2.5. If Y/PY/NI to 2.4: Were these deviations from intended intervention balanced between groups? | | | NA |  |
|  | 2.6 Was an appropriate analysis used to estimate the effect of assignment to intervention? | | | NI |  |
|  | 2.7 If N/PN/NI to 2.6: Was there potential for a substantial impact (on the result) of the failure to analyse participants in the group to which they were randomized? | | | NI |  |
|  | **Risk of bias judgement** | | | **High** | Blinding procedure is difficult to obtain |
| **Bias due to missing outcome data** | 3.1 Were data for this outcome available for all, or nearly all, participants randomized? | | | N | 18.8% loss to follow up at 5yrs (81 vs 87.5% between groups) |
|  | 3.2 If N/PN/NI to 3.1: Is there evidence that result was not biased by missing outcome data? | | | N |  |
|  | 3.3 If N/PN to 3.2: Could missingness in the outcome depend on its true value? | | | PY | It is reasonable to presume that patients lost to follow up in a bariatric study are lost due at least in part of poor postoperative outcomes, and that those not being followed up at five years will be different to those remaining in follow up. |
|  | 3.4 If Y/PY/NI to 3.3: Is it likely that missingness in the outcome depended on its true value? | | | PY |  |
|  | **Risk of bias judgement** | | | **High** |  |
| **Bias in measurement of the outcome** | 4.1 Was the method of measuring the outcome inappropriate? | | | N | Despite the lack of definition for GERD, all revisional surgeries and conversions should be picked up in the re-operation data. |
|  | 4.2 Could measurement or ascertainment of the outcome have differed between intervention groups? | | | PN | No - surgery would be comparable between the two procedures. |
|  | 4.3 Were outcome assessors aware of the intervention received by study participants? | | | Y | Surgeons would be aware of what surgery their patient had had. |
|  | 4.4 If Y/PY/NI to 4.3: Could assessment of the outcome have been influenced by knowledge of intervention received? | | | PN | Return to surgery would be guided by the severity of symptoms experienced therefore unlikely to be determined by prior knowledge of the procedure provided. |
|  | 4.5 If Y/PY/NI to 4.4: Is it likely that assessment of the outcome was influenced by knowledge of intervention received? | | | NA |  |
|  | **Risk of bias judgement** | | | **Low** |  |
| **Bias in selection of the reported result** | 5.1 Were the data that produced this result analysed in accordance with a pre-specified analysis plan that was finalized before unblinded outcome data were available for analysis? | | | N | Statistical analysis not stated as being determined prior to study, and no relationship to time prior to unblinding of analysts mentioned. |
|  | 5.2 ... multiple eligible outcome measurements (e.g. scales, definitions, time points) within the outcome domain? | | | PN | Without a definition of GERD, the measure is likely to be subjective. |
|  | 5.3 ... multiple eligible analyses of the data? | | | N | No |
|  | **Risk of bias judgement** | | | **Some concerns** |  |
| **Overall bias** | **Risk of bias judgement** | | | **High** | Measurement less of an issue than in other parameters but those lost to follow up |
|  |  |  |  |  |  |
|  |  |  |  |  |  |
| **Unique ID** | 4 | **Study ID** | 2 | **Assessor** | MAM/EO |
| **Author/Ref** | Ignat et al^15^ | **Aim** | assignment to intervention (the 'intention-to-treat' effect) |  |  |
| **Experimental** | LVSG | **Comparator** | LRYGB | **Source** | Journal article(s) |
| **Outcome** | Worsened and De Novo GERD | **Results** |  | **Weight** | 1 |
| **Domain** | **Signalling question** | | | **Response** | **Comments** |
| **Bias arising from the randomization process** | 1.1 Was the allocation sequence random? | | | PY | "The randomization was done using closed envelopes. For the 100 patients required, 120 envelopes were prepared, because of an estimated minimum 10 per cent failure rate after randomization (as a consequence of insurance reimbursement practices)".  Not stated but I would assume based on the intent to randomize this would not be identified until the time of procedure |
|  | 1.2 Was the allocation sequence concealed until participants were enrolled and assigned to interventions? | | | PY |  |
|  | 1.3 Did baseline differences between intervention groups suggest a problem with the randomization process? | | | PN | Table 1 appears roughly comparable, though not statistical test applied to these differences. |
|  | **Risk of bias judgement** | | | **Some concerns** |  |
| **Bias due to deviations from intended interventions** | 2.1.Were participants aware of their assigned intervention during the trial? | | | Y | Not blinded to participants or their families |
|  | 2.2.Were carers and people delivering the interventions aware of participants' assigned intervention during the trial? | | | Y |  |
|  | 2.3. If Y/PY/NI to 2.1 or 2.2: Were there deviations from the intended intervention that arose because of the experimental context? | | | PN |  |
|  | 2.4 If Y/PY to 2.3: Were these deviations likely to have affected the outcome? | | | NA |  |
|  | 2.5. If Y/PY/NI to 2.4: Were these deviations from intended intervention balanced between groups? | | | NA |  |
|  | 2.6 Was an appropriate analysis used to estimate the effect of assignment to intervention? | | | PY | quantitative reporting only |
|  | 2.7 If N/PN/NI to 2.6: Was there potential for a substantial impact (on the result) of the failure to analyse participants in the group to which they were randomized? | | | NA |  |
|  | **Risk of bias judgement** | | | **Low** |  |
| **Bias due to missing outcome data** | 3.1 Were data for this outcome available for all, or nearly all, participants randomized? | | | N | 28% loss to follow up at 5yrs, 7 pts (7.8%) had not completed 5yrs of data at time of publication |
|  | 3.2 If N/PN/NI to 3.1: Is there evidence that result was not biased by missing outcome data? | | | N | Highly likely to bias results |
|  | 3.3 If N/PN to 3.2: Could missingness in the outcome depend on its true value? | | | PY |  |
|  | 3.4 If Y/PY/NI to 3.3: Is it likely that missingness in the outcome depended on its true value? | | | PY |  |
|  | **Risk of bias judgement** | | | **High** |  |
| **Bias in measurement of the outcome** | 4.1 Was the method of measuring the outcome inappropriate? | | | N | Not defined as an adverse outcome. Based on criteria appears that would only report this if related to hospital admission |
|  | 4.2 Could measurement or ascertainment of the outcome have differed between intervention groups? | | | PN | Should be comparable between groups. |
|  | 4.3 Were outcome assessors aware of the intervention received by study participants? | | | Y | Surgeons would have to know what surgery the patient had |
|  | 4.4 If Y/PY/NI to 4.3: Could assessment of the outcome have been influenced by knowledge of intervention received? | | | PN | It was not a defined outcome and management should have been guided by presentation with severe symptoms (based on criteria) |
|  | 4.5 If Y/PY/NI to 4.4: Is it likely that assessment of the outcome was influenced by knowledge of intervention received? | | | NA |  |
|  | **Risk of bias judgement** | | | **Low** |  |
| **Bias in selection of the reported result** | 5.1 Were the data that produced this result analysed in accordance with a pre-specified analysis plan that was finalized before unblinded outcome data were available for analysis? | | | PN | Statistical plan was not indicated to be pre-specified and no indication of any blinding |
|  | 5.2 ... multiple eligible outcome measurements (e.g. scales, definitions, time points) within the outcome domain? | | | PN | No definition or description |
|  | 5.3 ... multiple eligible analyses of the data? | | | N |  |
|  | **Risk of bias judgement** | | | **Some concerns** |  |
| **Overall bias** | **Risk of bias judgement** | | | **High** | Primary concern related to lack of GERD data being specifically assessed outcome, more so that would only be picked up by reporting criteria if required hospital representation |
|  |  |  |  |  |  |
|  |  |  |  |  |  |
| **Unique ID** | 5 | **Study ID** | 2 | **Assessor** | MAM/EO |
| **Author/Ref** | Ignat et al^15^ | **Aim** | assignment to intervention (the 'intention-to-treat' effect) |  |  |
| **Experimental** | LVSG | **Comparator** | LRYGB | **Source** | Journal article(s) |
| **Outcome** | Increased or new need for Rx | **Results** |  | **Weight** | 1 |
| **Domain** | **Signalling question** | | | **Response** | **Comments** |
| **Bias arising from the randomization process** | 1.1 Was the allocation sequence random? | | | PY | "The randomization was done using closed envelopes. For the 100 patients required, 120 envelopes were prepared, because of an estimated minimum 10 per cent failure rate after randomization (as a consequence of insurance reimbursement practices)".  Not stated but I would assume based on the intent to randomize this would not be identified until the time of procedure |
|  | 1.2 Was the allocation sequence concealed until participants were enrolled and assigned to interventions? | | | PY |  |
|  | 1.3 Did baseline differences between intervention groups suggest a problem with the randomization process? | | | PN |  |
|  | **Risk of bias judgement** | | | **Low** |  |
| **Bias due to deviations from intended interventions** | 2.1.Were participants aware of their assigned intervention during the trial? | | | Y |  |
|  | 2.2.Were carers and people delivering the interventions aware of participants' assigned intervention during the trial? | | | Y |  |
|  | 2.3. If Y/PY/NI to 2.1 or 2.2: Were there deviations from the intended intervention that arose because of the experimental context? | | | PN |  |
|  | 2.4 If Y/PY to 2.3: Were these deviations likely to have affected the outcome? | | | NA |  |
|  | 2.5. If Y/PY/NI to 2.4: Were these deviations from intended intervention balanced between groups? | | | NA |  |
|  | 2.6 Was an appropriate analysis used to estimate the effect of assignment to intervention? | | | Y |  |
|  | 2.7 If N/PN/NI to 2.6: Was there potential for a substantial impact (on the result) of the failure to analyse participants in the group to which they were randomized? | | | NA |  |
|  | **Risk of bias judgement** | | | **Low** |  |
| **Bias due to missing outcome data** | 3.1 Were data for this outcome available for all, or nearly all, participants randomized? | | | N | 28% loss to follow up at 5yrs, 7 pts (7.8%) had not completed 5yrs of data at time of publication |
|  | 3.2 If N/PN/NI to 3.1: Is there evidence that result was not biased by missing outcome data? | | | N | Highly likely to bias results |
|  | 3.3 If N/PN to 3.2: Could missingness in the outcome depend on its true value? | | | PY |  |
|  | 3.4 If Y/PY/NI to 3.3: Is it likely that missingness in the outcome depended on its true value? | | | PY |  |
|  | **Risk of bias judgement** | | | **High** |  |
| **Bias in measurement of the outcome** | 4.1 Was the method of measuring the outcome inappropriate? | | | PN | Not defined as an adverse outcome. Based on criteria appears that would only report this if related to hospital admission |
|  | 4.2 Could measurement or ascertainment of the outcome have differed between intervention groups? | | | PN | Should be comparable between groups. |
|  | 4.3 Were outcome assessors aware of the intervention received by study participants? | | | Y | Surgeons would have to know what surgery the patient had |
|  | 4.4 If Y/PY/NI to 4.3: Could assessment of the outcome have been influenced by knowledge of intervention received? | | | PN | It was not a defined outcome and management should have been guided by presentation with severe symptoms (based on criteria) |
|  | 4.5 If Y/PY/NI to 4.4: Is it likely that assessment of the outcome was influenced by knowledge of intervention received? | | | NA |  |
|  | **Risk of bias judgement** | | | **Some concerns** |  |
| **Bias in selection of the reported result** | 5.1 Were the data that produced this result analysed in accordance with a pre-specified analysis plan that was finalized before unblinded outcome data were available for analysis? | | | PN | Statistical plan was not indicated to be pre-specified and no indication of any blinding |
|  | 5.2 ... multiple eligible outcome measurements (e.g. scales, definitions, time points) within the outcome domain? | | | PN | No definition or description |
|  | 5.3 ... multiple eligible analyses of the data? | | | N |  |
|  | **Risk of bias judgement** | | | **Some concerns** |  |
| **Overall bias** | **Risk of bias judgement** | | | **High** | Primary concern related to lack of GERD data being specifically assessed outcome, more so that would only be picked up by reporting criteria if required hospital representation |
|  |  |  |  |  |  |
|  |  |  |  |  |  |
| **Unique ID** | 6 | **Study ID** | 2 | **Assessor** | MAM/EO |
| **Author/Ref** | Ignat et al^15^ | **Aim** | assignment to intervention (the 'intention-to-treat' effect) |  |  |
| **Experimental** | LVSG | **Comparator** | LRYGB | **Source** | Journal article(s) |
| **Outcome** | Surgical interventino | **Results** |  | **Weight** | 1 |
| **Domain** | **Signalling question** | | | **Response** | **Comments** |
| **Bias arising from the randomization process** | 1.1 Was the allocation sequence random? | | | PY | "The randomization was done using closed envelopes. For the 100 patients required, 120 envelopes were prepared, because of an estimated minimum 10 per cent failure rate after randomization (as a consequenence of insurance reimbursement practices)".  Not stated but I would assume based on the intent to randomize this would not be identified until the time of procedure |
|  | 1.2 Was the allocation sequence concealed until participants were enrolled and assigned to interventions? | | | PY |  |
|  | 1.3 Did baseline differences between intervention groups suggest a problem with the randomization process? | | | PN |  |
|  | **Risk of bias judgement** | | | **Low** |  |
| **Bias due to deviations from intended interventions** | 2.1.Were participants aware of their assigned intervention during the trial? | | | Y |  |
|  | 2.2.Were carers and people delivering the interventions aware of participants' assigned intervention during the trial? | | | Y |  |
|  | 2.3. If Y/PY/NI to 2.1 or 2.2: Were there deviations from the intended intervention that arose because of the experimental context? | | | PN |  |
|  | 2.4 If Y/PY to 2.3: Were these deviations likely to have affected the outcome? | | | NA |  |
|  | 2.5. If Y/PY/NI to 2.4: Were these deviations from intended intervention balanced between groups? | | | NA |  |
|  | 2.6 Was an appropriate analysis used to estimate the effect of assignment to intervention? | | | PY | It is reasonable to assume that the need for surgical revision should result in readmission that would be captured in results |
|  | 2.7 If N/PN/NI to 2.6: Was there potential for a substantial impact (on the result) of the failure to analyse participants in the group to which they were randomized? | | | NA |  |
|  | **Risk of bias judgement** | | | **Low** |  |
| **Bias due to missing outcome data** | 3.1 Were data for this outcome available for all, or nearly all, participants randomized? | | | N | 28% loss to follow up at 5yrs, 7 pts (7.8%) had not completed 5yrs of data at time of publication |
|  | 3.2 If N/PN/NI to 3.1: Is there evidence that result was not biased by missing outcome data? | | | N | Highly likely to bias results |
|  | 3.3 If N/PN to 3.2: Could missingness in the outcome depend on its true value? | | | PY |  |
|  | 3.4 If Y/PY/NI to 3.3: Is it likely that missingness in the outcome depended on its true value? | | | PY |  |
|  | **Risk of bias judgement** | | | **High** |  |
| **Bias in measurement of the outcome** | 4.1 Was the method of measuring the outcome inappropriate? | | | PN | Not defined as an adverse outcome. Based on criteria appears that would only report this if related to hospital admission |
|  | 4.2 Could measurement or ascertainment of the outcome have differed between intervention groups? | | | PN | Should be comparable between groups. |
|  | 4.3 Were outcome assessors aware of the intervention received by study participants? | | | Y | Surgeons would have to know what surgery the patient had |
|  | 4.4 If Y/PY/NI to 4.3: Could assessment of the outcome have been influenced by knowledge of intervention received? | | | PN | It was not a defined outcome and management should have been guided by presentation with severe symptoms (based on criteria) |
|  | 4.5 If Y/PY/NI to 4.4: Is it likely that assessment of the outcome was influenced by knowledge of intervention received? | | | NA |  |
|  | **Risk of bias judgement** | | | **Low** |  |
| **Bias in selection of the reported result** | 5.1 Were the data that produced this result analysed in accordance with a pre-specified analysis plan that was finalized before unblinded outcome data were available for analysis? | | | PN | Statistical plan was not indicated to be pre-specified and no indication of any blinding |
|  | 5.2 ... multiple eligible outcome measurements (e.g. scales, definitions, time points) within the outcome domain? | | | PN | No definition or description |
|  | 5.3 ... multiple eligible analyses of the data? | | | N |  |
|  | **Risk of bias judgement** | | | **Some concerns** |  |
| **Overall bias** | **Risk of bias judgement** | | | **High** | Primary concern related to lack of GERD data being specifically assessed outcome, more so that would only be picked up by reporting criteria if required hospital representation |
|  |  |  |  |  |  |
|  |  |  |  |  |  |
| **Unique ID** | 7 | **Study ID** | 3 | **Assessor** | MAM/EO |
| **Author/Ref** | SLEEVEPASS^14^ | **Aim** | assignment to intervention (the 'intention-to-treat' effect) |  |  |
| **Experimental** | LVSG | **Comparator** | LRYGB | **Source** | Journal article(s) |
| **Outcome** | Worsened or de novo GERD | **Results** |  | **Weight** | 1 |
| **Domain** | **Signalling question** | | | **Response** | **Comments** |
| **Bias arising from the randomization process** | 1.1 Was the allocation sequence random? | | | Y | "Patients were randomized to by a closed envelope method to undergo either LSG or LRYGB. Randomization was performed with a 1:1 equal allocation ratio. The opaque, sealed, and sequentially number randomization envelopes were shuffled and then distributed to each participating hospital. To randomize an eligible patient after the clinical decision of proceeding to bariatric surgery for treatment of obesity, the surgeon opened a sealed envelope containing the information of the assigned randomization group." p242 |
|  | 1.2 Was the allocation sequence concealed until participants were enrolled and assigned to interventions? | | | Y |  |
|  | 1.3 Did baseline differences between intervention groups suggest a problem with the randomization process? | | | N | Table 1 indicates comparable groups p244 |
|  | **Risk of bias judgement** | | | **Low** |  |
| **Bias due to deviations from intended interventions** | 2.1.Were participants aware of their assigned intervention during the trial? | | | Y | Not overtly stated but this study was not blinded so it is safe to assume patients and their careers were informed by the surgeons which procedure they were randomized to. |
|  | 2.2.Were carers and people delivering the interventions aware of participants' assigned intervention during the trial? | | | Y |  |
|  | 2.3. If Y/PY/NI to 2.1 or 2.2: Were there deviations from the intended intervention that arose because of the experimental context? | | | PY | Fig 1 indicates 3 patients randomized to LRYGB didn't receive surgery (n=2) or didn't receive the surgery they were randomized (n=1) |
|  | 2.4 If Y/PY to 2.3: Were these deviations likely to have affected the outcome? | | | PN | Unlikely as low numbers representing 2.5% of LRYGB group |
|  | 2.5. If Y/PY/NI to 2.4: Were these deviations from intended intervention balanced between groups? | | | NA |  |
|  | 2.6 Was an appropriate analysis used to estimate the effect of assignment to intervention? | | | PY | the 1 patient who received LSG vs LRYGB was assess as LRYGB for ITT analysis |
|  | 2.7 If N/PN/NI to 2.6: Was there potential for a substantial impact (on the result) of the failure to analyse participants in the group to which they were randomized? | | | NA |  |
|  | **Risk of bias judgement** | | | **Some concerns** |  |
| **Bias due to missing outcome data** | 3.1 Were data for this outcome available for all, or nearly all, participants randomized? | | | N | 20% lost to follow up at 5yrs |
|  | 3.2 If N/PN/NI to 3.1: Is there evidence that result was not biased by missing outcome data? | | | N | no evidence that comparators between those lost to follow up and those in follow |
|  | 3.3 If N/PN to 3.2: Could missingness in the outcome depend on its true value? | | | PY | While not specific to GERD, patients may be lost to follow up because of worse outcomes or be in follow up to receive treatment for worse outcomes. |
|  | 3.4 If Y/PY/NI to 3.3: Is it likely that missingness in the outcome depended on its true value? | | | PY |  |
|  | **Risk of bias judgement** | | | **High** |  |
| **Bias in measurement of the outcome** | 4.1 Was the method of measuring the outcome inappropriate? | | | PN | GERD was assessed as complication and picked up under Clavien Dindo system for classification. Definition of GERD somewhat unclear and remains the biggest bias |
|  | 4.2 Could measurement or ascertainment of the outcome have differed between intervention groups? | | | PN | Similar issues existing for both group |
|  | 4.3 Were outcome assessors aware of the intervention received by study participants? | | | Y | Procedures not blinded to procedure |
|  | 4.4 If Y/PY/NI to 4.3: Could assessment of the outcome have been influenced by knowledge of intervention received? | | | PN | It is possible but not likely that knowledge of the procedure changed the GERD results reported. |
|  | 4.5 If Y/PY/NI to 4.4: Is it likely that assessment of the outcome was influenced by knowledge of intervention received? | | | NA |  |
|  | **Risk of bias judgement** | | | **Low** |  |
| **Bias in selection of the reported result** | 5.1 Were the data that produced this result analysed in accordance with a pre-specified analysis plan that was finalized before unblinded outcome data were available for analysis? | | | PY | Primary and secondary outcomes had predefined analysis plan; complications, under which GERD fall, was indicated to be assessed by Clavien Dindo. Frequency data reporting only |
|  | 5.2 ... multiple eligible outcome measurements (e.g. scales, definitions, time points) within the outcome domain? | | | N | Unlikely |
|  | 5.3 ... multiple eligible analyses of the data? | | | N | Unlikely |
|  | **Risk of bias judgement** | | | **Low** |  |
| **Overall bias** | **Risk of bias judgement** | | | **High** | Patients lost to follow up perhaps not representing whole is the largest bias for this outcome. Unclear GERD definition may also impact but likely mitigated by Clavien Dindo classification. GERD definitions not likely to be impacting on this outcome due to need for reoperation and prospective reporting of this. |
|  |  |  |  |  |  |
|  |  |  |  |  |  |
| **Unique ID** | 8 | **Study ID** | 3 | **Assessor** | MAM/EO |
| **Author/Ref** | SLEEVEPASS^14^ | **Aim** | assignment to intervention (the 'intention-to-treat' effect) |  |  |
| **Experimental** | LVSG | **Comparator** | LRYGB | **Source** | Journal article(s) |
| **Outcome** | Increased Rx management | **Results** |  | **Weight** | 1 |
| **Domain** | **Signalling question** | | | **Response** | **Comments** |
| **Bias arising from the randomization process** | 1.1 Was the allocation sequence random? | | | Y | "Patients were randomized to by a closed envelope method to undergo either LSG or LRYGB. Randomization was performed with a 1:1 equal allocation ratio. The opaque, sealed, and sequentially number randomization envelopes were shuffled and then distributed to each participating hospital. To randomize an eligible patient after the clinical decision of proceeding to bariatric surgery for treatment of obesity, the surgeon opened a sealed envelope containing the information of the assigned randomization group." p242 |
|  | 1.2 Was the allocation sequence concealed until participants were enrolled and assigned to interventions? | | | Y |  |
|  | 1.3 Did baseline differences between intervention groups suggest a problem with the randomization process? | | | N | Table 1 indicates comparable groups p244 |
|  | **Risk of bias judgement** | | | **Low** |  |
| **Bias due to deviations from intended interventions** | 2.1.Were participants aware of their assigned intervention during the trial? | | | Y | Not overtly stated but this study was not blinded so it is safe to assume patients and their careers were informed by the surgeons which procedure they were randomized to. |
|  | 2.2.Were carers and people delivering the interventions aware of participants' assigned intervention during the trial? | | | Y |  |
|  | 2.3. If Y/PY/NI to 2.1 or 2.2: Were there deviations from the intended intervention that arose because of the experimental context? | | | PY | Fig 1 indicates 3 patients randomized to LRYGB didn't receive surgery (n=2) or didn't receive the surgery they were randomized (n=1) |
|  | 2.4 If Y/PY to 2.3: Were these deviations likely to have affected the outcome? | | | PN | Unlikely as low numbers representing 2.5% of LRYGB group |
|  | 2.5. If Y/PY/NI to 2.4: Were these deviations from intended intervention balanced between groups? | | | NA |  |
|  | 2.6 Was an appropriate analysis used to estimate the effect of assignment to intervention? | | | PY | the 1 patient who received LSG vs LRYGB was assess as LRYGB for ITT analysis |
|  | 2.7 If N/PN/NI to 2.6: Was there potential for a substantial impact (on the result) of the failure to analyse participants in the group to which they were randomized? | | | NA |  |
|  | **Risk of bias judgement** | | | **Low** |  |
| **Bias due to missing outcome data** | 3.1 Were data for this outcome available for all, or nearly all, participants randomized? | | | N | 20% lost to follow up at 5yrs |
|  | 3.2 If N/PN/NI to 3.1: Is there evidence that result was not biased by missing outcome data? | | | N | no evidence that comparators between those lost to follow up and those in follow |
|  | 3.3 If N/PN to 3.2: Could missingness in the outcome depend on its true value? | | | PY | While not specific to GERD, patients may be lost to follow up because of worse outcomes or be in follow up to receive treatment for worse outcomes. |
|  | 3.4 If Y/PY/NI to 3.3: Is it likely that missingness in the outcome depended on its true value? | | | PY |  |
|  | **Risk of bias judgement** | | | **High** |  |
| **Bias in measurement of the outcome** | 4.1 Was the method of measuring the outcome inappropriate? | | | PN | GERD was assessed as complication and picked up under Clavien Dindo system for classification. Definition of GERD somewhat unclear and remains the biggest bias |
|  | 4.2 Could measurement or ascertainment of the outcome have differed between intervention groups? | | | PN | Similar issues existing for both group |
|  | 4.3 Were outcome assessors aware of the intervention received by study participants? | | | Y | Procedures not blinded to procedure |
|  | 4.4 If Y/PY/NI to 4.3: Could assessment of the outcome have been influenced by knowledge of intervention received? | | | PN | It is possible but not likely that knowledge of the procedure changed the GERD results reported. |
|  | 4.5 If Y/PY/NI to 4.4: Is it likely that assessment of the outcome was influenced by knowledge of intervention received? | | | NA |  |
|  | **Risk of bias judgement** | | | **Low** |  |
| **Bias in selection of the reported result** | 5.1 Were the data that produced this result analysed in accordance with a pre-specified analysis plan that was finalized before unblinded outcome data were available for analysis? | | | Y |  |
|  | 5.2 ... multiple eligible outcome measurements (e.g. scales, definitions, time points) within the outcome domain? | | | PN |  |
|  | 5.3 ... multiple eligible analyses of the data? | | | PN |  |
|  | **Risk of bias judgement** | | | **Low** |  |
| **Overall bias** | **Risk of bias judgement** | | | **High** | Patients lost to follow up perhaps not representing whole is the largest bias for this outcome. Unclear GERD definition may also impact but likely mitigated by Clavien Dindo classification. GERD definitions not likely to be impacting on this outcome due to need for reoperation and prospective reporting of this. |
|  |  |  |  |  |  |
|  |  |  |  |  |  |
| **Unique ID** | 9 | **Study ID** | 3 | **Assessor** | MAM/EO |
| **Author/Ref** | SLEEVEPASS^14^ | **Aim** | assignment to intervention (the 'intention-to-treat' effect) |  |  |
| **Experimental** | LVSG | **Comparator** | LRYGB | **Source** | Journal article(s) |
| **Outcome** | SURGICAL INTERVENTION FOR GERD | **Results** |  | **Weight** | 1 |
| **Domain** | **Signalling question** | | | **Response** | **Comments** |
| **Bias arising from the randomization process** | 1.1 Was the allocation sequence random? | | | Y | "Patients were randomized to by a closed envelope method to undergo either LSG or LRYGB. Randomization was performed with a 1:1 equal allocation ratio. The opaque, sealed, and sequentially number randomization envelopes were shuffled and then distributed to each participating hospital. To randomize an eligible patient after the clinical decision of proceeding to bariatric surgery for treatment of obesity, the surgeon opened a sealed envelope containing the information of the assigned randomization group." p242 |
|  | 1.2 Was the allocation sequence concealed until participants were enrolled and assigned to interventions? | | | Y |  |
|  | 1.3 Did baseline differences between intervention groups suggest a problem with the randomization process? | | | N | Table 1 indicates comparable groups p244 |
|  | **Risk of bias judgement** | | | **Low** |  |
| **Bias due to deviations from intended interventions** | 2.1.Were participants aware of their assigned intervention during the trial? | | | Y | Not overtly stated but this study was not blinded so it is safe to assume patients and their careers were informed by the surgeons which procedure they were randomized to. |
|  | 2.2.Were carers and people delivering the interventions aware of participants' assigned intervention during the trial? | | | Y |  |
|  | 2.3. If Y/PY/NI to 2.1 or 2.2: Were there deviations from the intended intervention that arose because of the experimental context? | | | PY | Fig 1 indicates 3 patients randomized to LRYGB didn't receive surgery (n=2) or didn't receive the surgery they were randomized (n=1) |
|  | 2.4 If Y/PY to 2.3: Were these deviations likely to have affected the outcome? | | | PN | Unlikely as low numbers representing 2.5% of LRYGB group |
|  | 2.5. If Y/PY/NI to 2.4: Were these deviations from intended intervention balanced between groups? | | | NA |  |
|  | 2.6 Was an appropriate analysis used to estimate the effect of assignment to intervention? | | | PY | the 1 patient who received LSG vs LRYGB was assess as LRYGB for ITT analysis |
|  | 2.7 If N/PN/NI to 2.6: Was there potential for a substantial impact (on the result) of the failure to analyse participants in the group to which they were randomized? | | | NA |  |
|  | **Risk of bias judgement** | | | **Some concerns** |  |
| **Bias due to missing outcome data** | 3.1 Were data for this outcome available for all, or nearly all, participants randomized? | | | N | 20% lost to follow up at 5yrs |
|  | 3.2 If N/PN/NI to 3.1: Is there evidence that result was not biased by missing outcome data? | | | N | no evidence that comparators between those lost to follow up and those in follow |
|  | 3.3 If N/PN to 3.2: Could missingness in the outcome depend on its true value? | | | PY | While not specific to GERD, patients may be lost to follow up because of worse outcomes or be in follow up to receive treatment for worse outcomes. |
|  | 3.4 If Y/PY/NI to 3.3: Is it likely that missingness in the outcome depended on its true value? | | | PY |  |
|  | **Risk of bias judgement** | | | **High** |  |
| **Bias in measurement of the outcome** | 4.1 Was the method of measuring the outcome inappropriate? | | | N | Reoperation was a reportable morbidity and therefore all surgical interventions associated with GERD symptoms would have been identified in patients remaining in follow up. |
|  | 4.2 Could measurement or ascertainment of the outcome have differed between intervention groups? | | | N | Comparable treatment of both groups. |
|  | 4.3 Were outcome assessors aware of the intervention received by study participants? | | | Y | Unblinded |
|  | 4.4 If Y/PY/NI to 4.3: Could assessment of the outcome have been influenced by knowledge of intervention received? | | | PN | Should not impact outcome of reoperation |
|  | 4.5 If Y/PY/NI to 4.4: Is it likely that assessment of the outcome was influenced by knowledge of intervention received? | | | NA |  |
|  | **Risk of bias judgement** | | | **Low** |  |
| **Bias in selection of the reported result** | 5.1 Were the data that produced this result analysed in accordance with a pre-specified analysis plan that was finalized before unblinded outcome data were available for analysis? | | | Y | Described as part of data collection and reporting plan |
|  | 5.2 ... multiple eligible outcome measurements (e.g. scales, definitions, time points) within the outcome domain? | | | N |  |
|  | 5.3 ... multiple eligible analyses of the data? | | | N |  |
|  | **Risk of bias judgement** | | | **Low** |  |
| **Overall bias** | **Risk of bias judgement** | | | **High** | Patients lost to follow up perhaps not representing whole is the largest bias for this outcome.  GERD definitions not likely to be impacting on this outcome due to need for reoperation and prospective reporting of this. |
|  |  |  |  |  |  |
|  |  |  |  |  |  |
| **Unique ID** | 10 | **Study ID** | 4 | **Assessor** | MAM/EO |
| **Author/Ref** | SMBOSS^13^ | **Aim** | assignment to intervention (the 'intention-to-treat' effect) |  |  |
| **Experimental** | LVSG | **Comparator** | LRYGB | **Source** | Journal article(s) |
| **Outcome** | Worsened or de novo GERD | **Results** |  | **Weight** | 1 |
| **Domain** | **Signalling question** | | | **Response** | **Comments** |
| **Bias arising from the randomization process** | 1.1 Was the allocation sequence random? | | | Y | "A central, computer-based block randomization (block size of 20) with sealed envelopes was carried out." p 256 |
|  | 1.2 Was the allocation sequence concealed until participants were enrolled and assigned to interventions? | | | PY |  |
|  | 1.3 Did baseline differences between intervention groups suggest a problem with the randomization process? | | | N | Comparable for GERD at baseline as per Table 1 |
|  | **Risk of bias judgement** | | | **Low** |  |
| **Bias due to deviations from intended interventions** | 2.1.Were participants aware of their assigned intervention during the trial? | | | Y | There was no blinding with regards to the type of operation: patients as well as physicians and dietitians assessing follow up data were informed about the procedure performed. |
|  | 2.2.Were carers and people delivering the interventions aware of participants' assigned intervention during the trial? | | | Y |  |
|  | 2.3. If Y/PY/NI to 2.1 or 2.2: Were there deviations from the intended intervention that arose because of the experimental context? | | | PN | 1 patient randomized to LRYGB received LVSG due to intraop technical difficulties. |
|  | 2.4 If Y/PY to 2.3: Were these deviations likely to have affected the outcome? | | | NA |  |
|  | 2.5. If Y/PY/NI to 2.4: Were these deviations from intended intervention balanced between groups? | | | NA |  |
|  | 2.6 Was an appropriate analysis used to estimate the effect of assignment to intervention? | | | PY | ITT analysis |
|  | 2.7 If N/PN/NI to 2.6: Was there potential for a substantial impact (on the result) of the failure to analyse participants in the group to which they were randomized? | | | NA |  |
|  | **Risk of bias judgement** | | | **Low** |  |
| **Bias due to missing outcome data** | 3.1 Were data for this outcome available for all, or nearly all, participants randomized? | | | PY | 8% loss to follow up at 5 yrs - comparable lost to follow-up between groups |
|  | 3.2 If N/PN/NI to 3.1: Is there evidence that result was not biased by missing outcome data? | | | NA |  |
|  | 3.3 If N/PN to 3.2: Could missingness in the outcome depend on its true value? | | | NA |  |
|  | 3.4 If Y/PY/NI to 3.3: Is it likely that missingness in the outcome depended on its true value? | | | NA |  |
|  | **Risk of bias judgement** | | | **Low** |  |
| **Bias in measurement of the outcome** | 4.1 Was the method of measuring the outcome inappropriate? | | | N | GERD described as a comorbidity in secondary outcomes. Some concerns by MAM regarding robustness of assessment of same however. |
|  | 4.2 Could measurement or ascertainment of the outcome have differed between intervention groups? | | | N | Comparable between groups in terms of process. |
|  | 4.3 Were outcome assessors aware of the intervention received by study participants? | | | Y | Not blinded as per page256 |
|  | 4.4 If Y/PY/NI to 4.3: Could assessment of the outcome have been influenced by knowledge of intervention received? | | | PN | Unlikely to affect outcome. |
|  | 4.5 If Y/PY/NI to 4.4: Is it likely that assessment of the outcome was influenced by knowledge of intervention received? | | | NA |  |
|  | **Risk of bias judgement** | | | **Low** |  |
| **Bias in selection of the reported result** | 5.1 Were the data that produced this result analysed in accordance with a pre-specified analysis plan that was finalized before unblinded outcome data were available for analysis? | | | Y | Analysis plan outlined with a view to obtain absolute difference. |
|  | 5.2 ... multiple eligible outcome measurements (e.g. scales, definitions, time points) within the outcome domain? | | | N |  |
|  | 5.3 ... multiple eligible analyses of the data? | | | N |  |
|  | **Risk of bias judgement** | | | **Low** |  |
| **Overall bias** | **Risk of bias judgement** | | | **Low** | Prospective review of GERD as comorbidity AND complication, best described definition and relatively low loss to follow up at 5 yrs (<10%) = low risk of bias. |
|  |  |  |  |  |  |
|  |  |  |  |  |  |
| **Unique ID** | 11 | **Study ID** | 4 | **Assessor** | MAM/EO |
| **Author/Ref** | SMBOSS^13^ | **Aim** | assignment to intervention (the 'intention-to-treat' effect) |  |  |
| **Experimental** | LVSG | **Comparator** | LRYGB | **Source** | Journal article(s) |
| **Outcome** | INCREASED RX | **Results** |  | **Weight** | 1 |
| **Domain** | **Signalling question** | | | **Response** | **Comments** |
| **Bias arising from the randomization process** | 1.1 Was the allocation sequence random? | | | Y | "A central, computer-based block randomization (block size of 20) with sealed envelopes was carried out." p 256 |
|  | 1.2 Was the allocation sequence concealed until participants were enrolled and assigned to interventions? | | | Y |  |
|  | 1.3 Did baseline differences between intervention groups suggest a problem with the randomization process? | | | N | Comparable for GERD at baseline as per Table 1 |
|  | **Risk of bias judgement** | | | **Low** |  |
| **Bias due to deviations from intended interventions** | 2.1.Were participants aware of their assigned intervention during the trial? | | | Y | There was no blinding with regard to the type of operation: patients as well as physicians and dietitians assessing follow up data were informed about the procedure performed. |
|  | 2.2.Were carers and people delivering the interventions aware of participants' assigned intervention during the trial? | | | Y |  |
|  | 2.3. If Y/PY/NI to 2.1 or 2.2: Were there deviations from the intended intervention that arose because of the experimental context? | | | PN | 1 patient randomized to LRYGB received LSG due to intraop technical difficulties. |
|  | 2.4 If Y/PY to 2.3: Were these deviations likely to have affected the outcome? | | | NA |  |
|  | 2.5. If Y/PY/NI to 2.4: Were these deviations from intended intervention balanced between groups? | | | NA |  |
|  | 2.6 Was an appropriate analysis used to estimate the effect of assignment to intervention? | | | Y | ITT analysis |
|  | 2.7 If N/PN/NI to 2.6: Was there potential for a substantial impact (on the result) of the failure to analyse participants in the group to which they were randomized? | | | NA |  |
|  | **Risk of bias judgement** | | | **Low** |  |
| **Bias due to missing outcome data** | 3.1 Were data for this outcome available for all, or nearly all, participants randomized? | | | PY | 8% loss to follow up at 5 yrs - comparable LTFU between groups |
|  | 3.2 If N/PN/NI to 3.1: Is there evidence that result was not biased by missing outcome data? | | | NA |  |
|  | 3.3 If N/PN to 3.2: Could missingness in the outcome depend on its true value? | | | NA |  |
|  | 3.4 If Y/PY/NI to 3.3: Is it likely that missingness in the outcome depended on its true value? | | | NA |  |
|  | **Risk of bias judgement** | | | **Low** |  |
| **Bias in measurement of the outcome** | 4.1 Was the method of measuring the outcome inappropriate? | | | N | GERD described as a comorbidity in secondary outcomes. Some concerns by MAM regarding robustness of assessment of same however. |
|  | 4.2 Could measurement or ascertainment of the outcome have differed between intervention groups? | | | N | Comparable between groups in terms of process. |
|  | 4.3 Were outcome assessors aware of the intervention received by study participants? | | | Y | Not blinded as per page256 |
|  | 4.4 If Y/PY/NI to 4.3: Could assessment of the outcome have been influenced by knowledge of intervention received? | | | PN | Unlikely to affect outcome. |
|  | 4.5 If Y/PY/NI to 4.4: Is it likely that assessment of the outcome was influenced by knowledge of intervention received? | | | NA |  |
|  | **Risk of bias judgement** | | | **Low** |  |
| **Bias in selection of the reported result** | 5.1 Were the data that produced this result analysed in accordance with a pre-specified analysis plan that was finalized before unblinded outcome data were available for analysis? | | | Y | Analysis plan outlined with a view to obtain absolute difference. |
|  | 5.2 ... multiple eligible outcome measurements (e.g. scales, definitions, time points) within the outcome domain? | | | N |  |
|  | 5.3 ... multiple eligible analyses of the data? | | | N |  |
|  | **Risk of bias judgement** | | | **Low** |  |
| **Overall bias** | **Risk of bias judgement** | | | **Low** | Prospective review of GERD as comorbidity AND complication, best described definition and relatively low loss to follow up at 5 yrs (<10%) = low risk of bias. |
|  |  |  |  |  |  |
|  |  |  |  |  |  |
| **Unique ID** | 12 | **Study ID** | 4 | **Assessor** | MAM/EO |
| **Author/Ref** | SMBOSS^13^ | **Aim** | assignment to intervention (the 'intention-to-treat' effect) |  |  |
| **Experimental** | LVSG | **Comparator** | LRYGB | **Source** | Journal article(s) |
| **Outcome** | REOPERATIONS FOR GERD | **Results** |  | **Weight** | 1 |
| **Domain** | **Signalling question** | | | **Response** | **Comments** |
| **Bias arising from the randomization process** | 1.1 Was the allocation sequence random? | | | Y | "A central, computer-based block randomization (block size of 20) with sealed envelopes was carried out." p 256 |
|  | 1.2 Was the allocation sequence concealed until participants were enrolled and assigned to interventions? | | | Y |  |
|  | 1.3 Did baseline differences between intervention groups suggest a problem with the randomization process? | | | N | Comparable for GERD at baseline as per Table 1 |
|  | **Risk of bias judgement** | | | **Low** |  |
| **Bias due to deviations from intended interventions** | 2.1.Were participants aware of their assigned intervention during the trial? | | | Y | There was no blinding with regard to the type of operation: patients as well as physicians and dietitians assessing follow up data were informed about the procedure performed. |
|  | 2.2.Were carers and people delivering the interventions aware of participants' assigned intervention during the trial? | | | Y |  |
|  | 2.3. If Y/PY/NI to 2.1 or 2.2: Were there deviations from the intended intervention that arose because of the experimental context? | | | PN | 1 patient randomized to LRYGB received LSG due to intraop technical difficulties. |
|  | 2.4 If Y/PY to 2.3: Were these deviations likely to have affected the outcome? | | | NA |  |
|  | 2.5. If Y/PY/NI to 2.4: Were these deviations from intended intervention balanced between groups? | | | NA |  |
|  | 2.6 Was an appropriate analysis used to estimate the effect of assignment to intervention? | | | PY | ITT analysis |
|  | 2.7 If N/PN/NI to 2.6: Was there potential for a substantial impact (on the result) of the failure to analyse participants in the group to which they were randomized? | | | NA |  |
|  | **Risk of bias judgement** | | | **Low** |  |
| **Bias due to missing outcome data** | 3.1 Were data for this outcome available for all, or nearly all, participants randomized? | | | PY | 8% loss to follow up at 5 yrs - comparable LTFU between groups |
|  | 3.2 If N/PN/NI to 3.1: Is there evidence that result was not biased by missing outcome data? | | | NA |  |
|  | 3.3 If N/PN to 3.2: Could missingness in the outcome depend on its true value? | | | NA |  |
|  | 3.4 If Y/PY/NI to 3.3: Is it likely that missingness in the outcome depended on its true value? | | | NA |  |
|  | **Risk of bias judgement** | | | **Low** |  |
| **Bias in measurement of the outcome** | 4.1 Was the method of measuring the outcome inappropriate? | | | N | GERD described as a comorbidity in secondary outcomes. Some concerns by MAM regarding robustness of assessment of same however. |
|  | 4.2 Could measurement or ascertainment of the outcome have differed between intervention groups? | | | N | Comparable between groups in terms of process. |
|  | 4.3 Were outcome assessors aware of the intervention received by study participants? | | | Y | Not blinded as per page256 |
|  | 4.4 If Y/PY/NI to 4.3: Could assessment of the outcome have been influenced by knowledge of intervention received? | | | PN | Unlikely to affect outcome. |
|  | 4.5 If Y/PY/NI to 4.4: Is it likely that assessment of the outcome was influenced by knowledge of intervention received? | | | NA |  |
|  | **Risk of bias judgement** | | | **Low** |  |
| **Bias in selection of the reported result** | 5.1 Were the data that produced this result analysed in accordance with a pre-specified analysis plan that was finalized before unblinded outcome data were available for analysis? | | | Y | Analysis plan outlined with a view to obtain absolute difference. |
|  | 5.2 ... multiple eligible outcome measurements (e.g. scales, definitions, time points) within the outcome domain? | | | N |  |
|  | 5.3 ... multiple eligible analyses of the data? | | | PN |  |
|  | **Risk of bias judgement** | | | **Low** |  |
| **Overall bias** | **Risk of bias judgement** | | | **Low** | Prospective review of GERD as comorbidity AND complication, best described definition and relatively low loss to follow up at 5 yrs (<10%) = low risk of bias. |

| **Unique ID** | 13 | **Study ID** | 5 | **Assessor** | MAM/EO |
| --- | --- | --- | --- | --- | --- |
| **Author/Ref** | SleeveBypass^16^ | **Aim** | assignment to intervention (the 'intention-to-treat' effect) |  |  |
| **Experimental** | LVSG | **Comparator** | LRYGB | **Source** | Journal article(s) |
| **Outcome** | Worsened or de novo GERD | **Results** |  | **Weight** | 1 |
| **Domain** | **Signalling question** | | | **Response** | **Comments** |
| **Bias arising from the randomization process** | 1.1 Was the allocation sequence random? | | | Y | " Randomization 1:1 using computer variable block randomization (block sizes: 6,8,12)" (p3). |
|  | 1.2 Was the allocation sequence concealed until participants were enrolled and assigned to interventions? | | | Y |  |
|  | 1.3 Did baseline differences between intervention groups suggest a problem with the randomization process? | | | N | Comparable groups for demographics and preop co-morbidities (table 1). Preop GERD outcomes described. |
|  | **Risk of bias judgement** | | | **Low** |  |
| **Bias due to deviations from intended interventions** | 2.1.Were participants aware of their assigned intervention during the trial? | | | Y | No masking of investigators including primary outcome assessor or patients to treatment allocation was performed |
|  | 2.2.Were carers and people delivering the interventions aware of participants' assigned intervention during the trial? | | | Y |  |
|  | 2.3. If Y/PY/NI to 2.1 or 2.2: Were there deviations from the intended intervention that arose because of the experimental context? | | | PN | Nil reported |
|  | 2.4 If Y/PY to 2.3: Were these deviations likely to have affected the outcome? | | | NA |  |
|  | 2.5. If Y/PY/NI to 2.4: Were these deviations from intended intervention balanced between groups? | | | NA |  |
|  | 2.6 Was an appropriate analysis used to estimate the effect of assignment to intervention? | | | Y | Based on previous protocol published in BMC obesity |
|  | 2.7 If N/PN/NI to 2.6: Was there potential for a substantial impact (on the result) of the failure to analyse participants in the group to which they were randomized? | | | NI |  |
|  | **Risk of bias judgement** | | | **High** | Blinding procedure is difficult to obtain |
| **Bias due to missing outcome data** | 3.1 Were data for this outcome available for all, or nearly all, participants randomized? | | | PN | 22.7% loss to follow up at 5yrs (79.1% vs 75.6% LVSG vs LRYGB) |
|  | 3.2 If N/PN/NI to 3.1: Is there evidence that result was not biased by missing outcome data? | | | PN |  |
|  | 3.3 If N/PN to 3.2: Could missingness in the outcome depend on its true value? | | | PY | It is reasonable to presume that patients lost to follow up in a bariatric study are lost due at least in part of poor postoperative outcomes, and that those not being followed up at five years will be different to those remaining in follow up. |
|  | 3.4 If Y/PY/NI to 3.3: Is it likely that missingness in the outcome depended on its true value? | | | PY |  |
|  | **Risk of bias judgement** | | | **High** |  |
| **Bias in measurement of the outcome** | 4.1 Was the method of measuring the outcome inappropriate? | | | PN | GERD is identified based on subjective criteria and on priori outcome. Postop improvement/deterioration in GERD symptoms and increase/decreased in pharmacotherapy along with new onset GERD was based on subjective criteria |
|  | 4.2 Could measurement or ascertainment of the outcome have differed between intervention groups? | | | PY | Without a clear GERD definition, detection may vary between clinicians based on subjective criteria. |
|  | 4.3 Were outcome assessors aware of the intervention received by study participants? | | | Y |  |
|  | 4.4 If Y/PY/NI to 4.3: Could assessment of the outcome have been influenced by knowledge of intervention received? | | | Y | Prior surgical intervention knowledge plays an important role in the analysis of subjective data by the outcome assessors |
|  | 4.5 If Y/PY/NI to 4.4: Is it likely that assessment of the outcome was influenced by knowledge of intervention received? | | | Y |  |
|  | **Risk of bias judgement** | | | **High** |  |
| **Bias in selection of the reported result** | 5.1 Were the data that produced this result analysed in accordance with a pre-specified analysis plan that was finalized before unblinded outcome data were available for analysis? | | | PY | No blinding included in the methodology, so results cannot be unblinded irrespective of pre-published protocol.  Statistical analysis plan was published prior to study (see protocol in 2015 BMC Obesity), Sample size determination and %EBMIL was determined but not specifically for any GERD outcomes |
|  | 5.2 ... multiple eligible outcome measurements (e.g. scales, definitions, time points) within the outcome domain? | | | PN | Without an objective definition of GERD, the measure is likely to be subjective. |
|  | 5.3 ... multiple eligible analyses of the data? | | | N | No |
|  | **Risk of bias judgement** | | | **Some concerns** |  |
| **Overall bias** | **Risk of bias judgement** | | | **High** | Main issue stems from subjective definition of GERD preop and no objective measurement via 24 hr pH study. Postop assessment also based on patients’ questionnaire without any objective assessment |
|  |  |  |  |  |  |
|  |  |  |  |  |  |
| **Unique ID** | 14 | **Study ID** | 5 | **Assessor** | MAM/EO |
| **Author/Ref** | SleeveBypass^16^ | **Aim** | assignment to intervention (the 'intention-to-treat' effect) |  |  |
| **Experimental** | LVSG | **Comparator** | LRGB | **Source** | Journal article(s) |
| **Outcome** | Increased or new need for Rx | **Results** |  | **Weight** | 1 |
| **Domain** | **Signalling question** | | | **Response** | **Comments** |
| **Bias arising from the randomization process** | 1.1 Was the allocation sequence random? | | | Y | " Randomization 1:1 using computer variable block randomization (block sizes: 6,8,12)" (p3). |
|  | 1.2 Was the allocation sequence concealed until participants were enrolled and assigned to interventions? | | | Y |  |
|  | 1.3 Did baseline differences between intervention groups suggest a problem with the randomization process? | | | N | Comparable groups for demographics and preop co-morbidities (table 1). Preop GERD outcomes described. |
|  | **Risk of bias judgement** | | | **Low** |  |
| **Bias due to deviations from intended interventions** | 2.1.Were participants aware of their assigned intervention during the trial? | | | Y | No masking of investigators including primary outcome assessor or patients to treatment allocation was performed |
|  | 2.2.Were carers and people delivering the interventions aware of participants' assigned intervention during the trial? | | | Y |  |
|  | 2.3. If Y/PY/NI to 2.1 or 2.2: Were there deviations from the intended intervention that arose because of the experimental context? | | | PN | Nil reported |
|  | 2.4 If Y/PY to 2.3: Were these deviations likely to have affected the outcome? | | | NA |  |
|  | 2.5. If Y/PY/NI to 2.4: Were these deviations from intended intervention balanced between groups? | | | NA |  |
|  | 2.6 Was an appropriate analysis used to estimate the effect of assignment to intervention? | | | Y |  |
|  | 2.7 If N/PN/NI to 2.6: Was there potential for a substantial impact (on the result) of the failure to analyse participants in the group to which they were randomized? | | | NI |  |
|  | **Risk of bias judgement** | | | **High** | Blinding procedure is difficult to obtain |
| **Bias due to missing outcome data** | 3.1 Were data for this outcome available for all, or nearly all, participants randomized? | | | PN | 22.7% loss to follow up at 5yrs (79.1% vs 75.6% LVSG vs LRYGB) |
|  | 3.2 If N/PN/NI to 3.1: Is there evidence that result was not biased by missing outcome data? | | | PN |  |
|  | 3.3 If N/PN to 3.2: Could missingness in the outcome depend on its true value? | | | PY | It is reasonable to presume that patients lost to follow up in a bariatric study are lost due at least in part of poor postoperative outcomes, and that those not being followed up at five years will be different to those remaining in follow up. |
|  | 3.4 If Y/PY/NI to 3.3: Is it likely that missingness in the outcome depended on its true value? | | | PY |  |
|  | **Risk of bias judgement** | | | **High** |  |
| **Bias in measurement of the outcome** | 4.1 Was the method of measuring the outcome inappropriate? | | | PN | GERD is identified based on subjective criteria and on priori outcome. Postop improvement/deterioration in GERD symptoms and increase/decreased in pharmacotherapy along with new onset GERD was based on subjective criteria |
|  | 4.2 Could measurement or ascertainment of the outcome have differed between intervention groups? | | | PY | Without a clear GERD definition, detection may vary between clinicians based on subjective criteria. |
|  | 4.3 Were outcome assessors aware of the intervention received by study participants? | | | Y |  |
|  | 4.4 If Y/PY/NI to 4.3: Could assessment of the outcome have been influenced by knowledge of intervention received? | | | Y |  |
|  | 4.5 If Y/PY/NI to 4.4: Is it likely that assessment of the outcome was influenced by knowledge of intervention received? | | | Y |  |
|  | **Risk of bias judgement** | | | **High** |  |
| **Bias in selection of the reported result** | 5.1 Were the data that produced this result analysed in accordance with a pre-specified analysis plan that was finalized before unblinded outcome data were available for analysis? | | | PY | No blinding included in the methodology, so results cannot be unblinded irrespective of pre-published protocol.  Statistical analysis was performed prior to study (see protocol published in 2015 BMC Obesity), Sample size determination and %EBMIL was determined but not specifically for any GERD outcomes |
|  | 5.2 ... multiple eligible outcome measurements (e.g. scales, definitions, time points) within the outcome domain? | | | PN | Without a definition of GERD, the measure is likely to be subjective. |
|  | 5.3 ... multiple eligible analyses of the data? | | | N | No |
|  | **Risk of bias judgement** | | | **Some concerns** |  |
| **Overall bias** | **Risk of bias judgement** | | | **High** | Main issue stems from subjective definition of GERD preop and no objective measurement via 24 hr pH study. Postop assessment also based on patients’ questionnaire without any objective assessment |
|  |  |  |  |  |  |
|  |  |  |  |  |  |
| **Unique ID** | 15 | **Study ID** | 5 | **Assessor** | MAM/EO |
| **Author/Ref** | SleeveBypass^16^ | **Aim** | assignment to intervention (the 'intention-to-treat' effect) |  |  |
| **Experimental** | LVSG | **Comparator** | LRGB | **Source** | Journal article(s) |
| **Outcome** | Surgical procedure required | **Results** |  | **Weight** | 1 |
| **Domain** | **Signalling question** | | | **Response** | **Comments** |
| **Bias arising from the randomization process** | 1.1 Was the allocation sequence random? | | | Y | " Randomization 1:1 using computer variable block randomization (block sizes: 6,8,12)" (p3). |
|  | 1.2 Was the allocation sequence concealed until participants were enrolled and assigned to interventions? | | | Y |  |
|  | 1.3 Did baseline differences between intervention groups suggest a problem with the randomization process? | | | N | Comparable groups for demographics and preop co-morbidities (table 1). Preop GERD outcomes described. |
|  | **Risk of bias judgement** | | | **Low** |  |
| **Bias due to deviations from intended interventions** | 2.1.Were participants aware of their assigned intervention during the trial? | | | Y | No masking of investigators including primary outcome assessor or patients to treatment allocation was performed |
|  | 2.2.Were carers and people delivering the interventions aware of participants' assigned intervention during the trial? | | | Y |  |
|  | 2.3. If Y/PY/NI to 2.1 or 2.2: Were there deviations from the intended intervention that arose because of the experimental context? | | | PN | Nil reported |
|  | 2.4 If Y/PY to 2.3: Were these deviations likely to have affected the outcome? | | | NA |  |
|  | 2.5. If Y/PY/NI to 2.4: Were these deviations from intended intervention balanced between groups? | | | NA |  |
|  | 2.6 Was an appropriate analysis used to estimate the effect of assignment to intervention? | | | Y |  |
|  | 2.7 If N/PN/NI to 2.6: Was there potential for a substantial impact (on the result) of the failure to analyse participants in the group to which they were randomized? | | | NI |  |
|  | **Risk of bias judgement** | | | **High** | Blinding procedure is difficult to obtain |
| **Bias due to missing outcome data** | 3.1 Were data for this outcome available for all, or nearly all, participants randomized? | | | PN | 22.7% loss to follow up at 5yrs (79.1 vs 75.6% LVSG vs LRYGB) |
|  | 3.2 If N/PN/NI to 3.1: Is there evidence that result was not biased by missing outcome data? | | | PN |  |
|  | 3.3 If N/PN to 3.2: Could missingness in the outcome depend on its true value? | | | PY | It is reasonable to presume that patients lost to follow up in a bariatric study are lost due at least in part of poor postoperative outcomes, and that those not being followed up at five years will be different to those remaining in follow up. |
|  | 3.4 If Y/PY/NI to 3.3: Is it likely that missingness in the outcome depended on its true value? | | | PY |  |
|  | **Risk of bias judgement** | | | **High** |  |
| **Bias in measurement of the outcome** | 4.1 Was the method of measuring the outcome inappropriate? | | | N | GERD is identified based on subjective criteria and on priori outcome. Postop improvement/deterioration in GERD symptoms and increase/decreased in pharmacotherapy along with new onset GERD was based on subjective criteria |
|  | 4.2 Could measurement or ascertainment of the outcome have differed between intervention groups? | | | PY | Surgery would be comparable between the two procedures. |
|  | 4.3 Were outcome assessors aware of the intervention received by study participants? | | | Y | Surgeons would be aware of what surgery their patient had had. |
|  | 4.4 If Y/PY/NI to 4.3: Could assessment of the outcome have been influenced by knowledge of intervention received? | | | Y | Return to surgery would be guided by the severity of symptoms experienced by the patient. However, no postoperative objective assessment following primary bariatric procedure was undertaken such as 24-hr pH study or esophageal manometry to assess these symptoms and therefore it is likely that prior knowledge of the procedure and surgeon’s skill set may have biased the opinion regarding type and timing of revisional surgery. |
|  | 4.5 If Y/PY/NI to 4.4: Is it likely that assessment of the outcome was influenced by knowledge of intervention received? | | | Y |  |
|  | **Risk of bias judgement** | | | **High** |  |
| **Bias in selection of the reported result** | 5.1 Were the data that produced this result analysed in accordance with a pre-specified analysis plan that was finalized before unblinded outcome data were available for analysis? | | | PY | No blinding included in the methodology, so results cannot be unblinded irrespective of pre-published protocol.  Statistical analysis was performed prior to study (see protocol published in 2015 BMC Obesity), Sample size determination and %EBMIL was determined but not specifically for any GERD outcomes |
|  | 5.2 ... multiple eligible outcome measurements (e.g. scales, definitions, time points) within the outcome domain? | | | PN | Without a definition of GERD, the measure is likely to be subjective. |
|  | 5.3 ... multiple eligible analyses of the data? | | | N | No |
|  | **Risk of bias judgement** | | | **Major concerns** |  |
| **Overall bias** | **Risk of bias judgement** | | | **High** | Main issue stems from subjective definition of GERD preop and no objective measurement via 24 hr pH study. Postop assessment also based on patients’ questionnaire without any objective assessment |
